# Parameter optimization by using differential elimination: a general approach for introducing constraints into objective functions

**DOI:** 10.1186/1752-0509-4-S2-S9

**Published:** 2010-09-13

**Authors:** Masahiko Nakatsui, Katsuhisa Horimoto, Masahiro Okamoto, Yasuhito Tokumoto, Jun Miyake

**Affiliations:** 1 Computational Biology Research Center, National Institute of Advanced Industrial Science and Technology, 2-4-7 Aomi, Koto-ku, Tokyo 135-0064, Japan; 2 Institute of Systems Biology, Shanghai University, Shangda Road 99, Shanghai 200444, China; 3 Laboratory for Bioinformatics, Graduate School of Systems Life Sciences, Kyushu University, 6-10-1 Hakozaki, Higashi-ku, Fukuoka 812-8581, Japan; 4 Department of Bioengineering, Graduate School of Engineering, The University of Tokyo, Tokyo 113-8656, Japan; 5 Department of Mechanical Science and Bioengineering, Graduate School of Engineering Science, Osaka University, Osaka 560-8531, Japan

## Abstract

**Background:**

The investigation of network dynamics is a major issue in systems and synthetic biology. One of the essential steps in a dynamics investigation is the parameter estimation in the model that expresses biological phenomena. Indeed, various techniques for parameter optimization have been devised and implemented in both free and commercial software. While the computational time for parameter estimation has been greatly reduced, due to improvements in calculation algorithms and the advent of high performance computers, the accuracy of parameter estimation has not been addressed.

**Results:**

We propose a new approach for parameter optimization by using differential elimination, to estimate kinetic parameter values with a high degree of accuracy. First, we utilize differential elimination, which is an algebraic approach for rewriting a system of differential equations into another equivalent system, to derive the constraints between kinetic parameters from differential equations. Second, we estimate the kinetic parameters introducing these constraints into an objective function, in addition to the error function of the square difference between the measured and estimated data, in the standard parameter optimization method. To evaluate the ability of our method, we performed a simulation study by using the objective function with and without the newly developed constraints: the parameters in two models of linear and non-linear equations, under the assumption that only one molecule in each model can be measured, were estimated by using a genetic algorithm (GA) and particle swarm optimization (PSO). As a result, the introduction of new constraints was dramatically effective: the GA and PSO with new constraints could successfully estimate the kinetic parameters in the simulated models, with a high degree of accuracy, while the conventional GA and PSO methods without them frequently failed.

**Conclusions:**

The introduction of new constraints in an objective function by using differential elimination resulted in the drastic improvement of the estimation accuracy in parameter optimization methods. The performance of our approach was illustrated by simulations of the parameter optimization for two models of linear and non-linear equations, which included unmeasured molecules, by two types of optimization techniques. As a result, our method is a promising development in parameter optimization.

## Background

The investigation of network dynamics is a major issue in systems and synthetic biology [1]. In general, a network model for describing the kinetics of constituent molecules is first constructed with reference to the biological knowledge, and then the model is mathematically expressed by differential equations, based on the chemical reactions underlying the kinetics. Finally, the kinetic parameters in the model are estimated by various parameter optimization techniques [2], from the time-series data measured for the constituent molecules. While the computational time for parameter estimation has been greatly reduced, due to the improvement in calculation algorithms and the advent of high performance computers, the accurate numerical estimation of parameter values for a given model remains a limiting step. Indeed, the parameter values estimated by various optimization techniques are frequently quite variable, due to the conditions for parameter estimation, such as the initial values. In particular, we cannot always obtain the data measured for all of the constituent molecules, due to limitations of measurement techniques and ethical constraints. In this case, one of the issues we should resolve is that the parameters are estimated from the data for only some of the constituent molecules. Unfortunately, it is quite difficult to estimate the parameters in such a network model including unmeasured variables.

Boulier and his colleagues developed differential elimination [3], derived from the Roselfeld-Gröbner base [4].  Differential elimination rewrites a system of original differential equations into an equivalent system. The rewriting feature was applied to solve the parameter optimization issue, especially in network dynamics including unmeasured variables [3,5], and  in the applications, the equations rewritten by differential elimination were utilized to estimate the initial values for the parameter optimization, by Newton-type numerical optimization.

Here, we propose a new method for optimizing the parameters, by using differential elimination [3]. Our method partially utilizes a technique from a previous study [3], regarding the introduction of differential elimination into parameter optimization in a network including unmeasured variables. Instead of using differential elimination for estimating the initial values for the following parameter optimization, the equations derived by differential elimination are directly introduced as the constraints into the objective function for the parameter optimization.  To validate the effectiveness of the constraint introduction, we performed simulations in two models of linear and nonlinear differential equations, where we assumed that the data for only one molecule among them were measured, by using two kinds of evolutionary optimization techniques. The accuracy of the parameter values estimated by the objective functions with and without the new constraints was compared. Finally, we discussed merits and pitfalls of our method in terms of its extension to more realistic and complex models. 

## Results 

We first describe a perspective of our method, and then the two models are analyzed to illustrate its performance. The two models were chosen from representative kinetic models for biological phenomena at the molecular level: one model (Model 1) is composed of two variables, analogous to molecular binding and dissociation, such as affinity binding in an antibody cross-link, and the other model (Model 2) is composed of four variables, analogous to a molecular reaction cascade, such as phosphorylation in signal transduction. Notably, we assumed that only one variable is measured among the variables in the two models. 

### Overview of present method

The key point of this study is the introduction of new constraints obtained by differential elimination into the objective function, to improve the parameter accuracy. Following an explanation of differential elimination, the method of introducing the constraints is briefly described.

Differential algebra aims at studying differential equations from a purely algebraic point of view [6,7]. Differential elimination theory is a sub theory of differential algebra [3], based on Rosenfeld-Gröbner [4]. The differential elimination rewrites the inputted system of differential equations to another equivalent system according to ranking (order of terms). Here, we provide an example of differential elimination, as shown below, according to Boulier [3,5].

**Figure 1:**
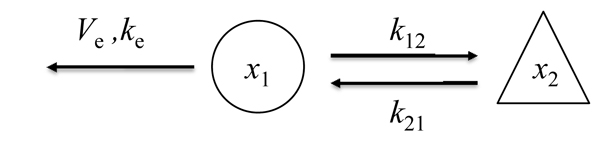
**Example model** Two molecules bind according to Michaelis-Menten kinetics, and only one molecule, *x*_1_, can be measured.

Assume a model of two variables, *x*_1_ and *x*_2_, in Fig. 1, which is described by the following system of parametric ordinary differential equations,

,								(1)

where *k*_12_, *k*_21_, *k*_e_ and *V*_e_ are some constants. Here, two molecules are assumed to bind according to Michaelis-Menten kinetics. The differential elimination then produces the following two equations equivalent to the above system.

			(2)

When we define the left sides of the above system as *C*_1,t_ and *C*_2,t_, *C*_2,t_ is composed of *x*_1_, its derivatives, and the parameters obtained by eliminating *x*_2_, and *C*_1,t_ is composed of *x*_1_, its derivatives, the parameters and *x*_2_. Note that *x*_2_ in *C*_1,t_ can be expressed by *x*_1_, its derivatives and the parameters in *C*_2,t_. Then, the values of *C*_1,t_ and *C*_2,t_ can be calculated, if we have time-series data of *x*_1_, and they would be zero, if all parameters were exactly estimated. Thus, *C*_1,t_ and *C*_2,t_ can be regarded as a kind of error function that expresses the difference between the measured and estimated data. 

In general, the typical objective function for evaluating the reproducibility of an experimentally measured time-series for a parameter set is the total relative error, *E*. The parameter set is then estimated when the total relative error falls below a given threshold. However, the immense searching space of parameter values frequently hinders correct parameter estimation. To overcome this problem, we introduce the constraint between the estimate obtained by differential elimination (DE constraints), *C*, into the objective function, i.e.,

,									(3)

where *α* is a weighting factor, which is approximately estimated by Pareto optimal solutions for *E* and *C*, and then is manually modified (see details in Methods). 

### Model 1

**Figure 2:**
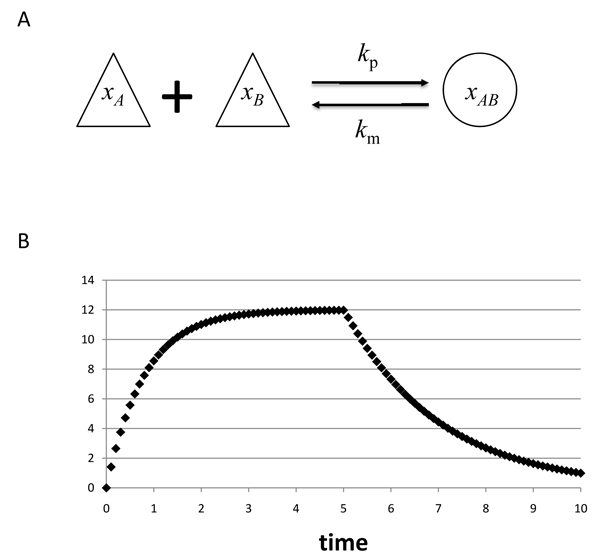
**Model 1: binding and dissociation.** The molecular binding and dissociation of two molecules is schematically shown (A). According to the kinetics of the model (see details in Methods), a reference curve of one variable, *x*_AB_, was generated for 0≦*t*≦1 with intervals of 0.01, under the following conditions: *x*_A_(0) = 10.0, *x*_B_(0) = 20.0, *x*_AB_(0) = 0.0, *k*_p_ = 0.05, *k*_m_ = 0.5, and *t*_c_=5.0 (B).

We analyzed a network model for the binding and dissociation of two molecules (Figure 2A). According to the kinetics of the model (see also Methods), the reference curve of one variable, *x*_AB_, was generated (Figure 2B), and two optimization techniques, genetic algorithm (GA) and particle swarm optimization (PSO), were applied to it to evaluate the effect of the introduction of differential elimination constraints (DE constraints) (see details in Additional File 1) into the objective function. 

**Figure 3:**
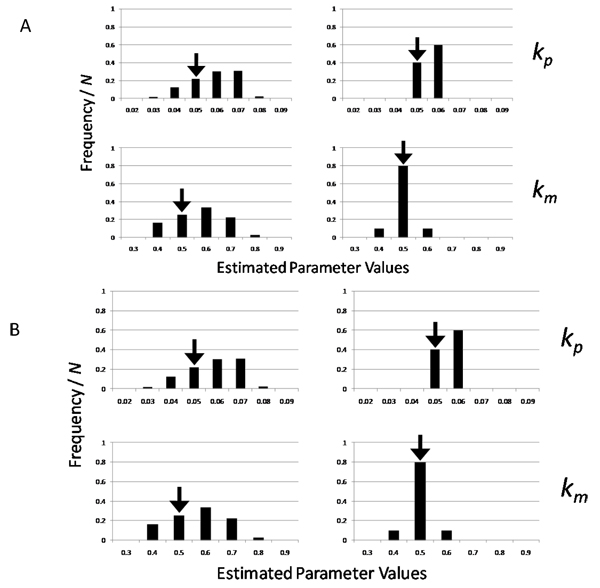
**Estimated parameter values for Model 1** The parameter sets are estimated by using the genetic algorithm (GA) (A) and the particle swarm optimization (PSO) (B), and in each figure, the histograms of parameter sets with and without DE constraints (right and left sides, respectively) are shown. The bin of the histogram indicates the fraction of the number of parameters within a range (0.01 for *k_p_* and 0.1 for *k_m_*) to the total number of trial successes (200 in GA and PSO without DE constraints, and 51 and 11 in GA and PSO with DE constraints, respectively) (see details in Methods).

Overall, the introduction of DE constraints into the objective function was highly effective for correctly estimating the parameter values in both GA and PSO (Figure 3). By using GA (Figure 3A), *k_p_* and km were correctly estimated with the introduction, while the estimation of *k_p_* failed without the introduction. Indeed, the most frequent values estimated with the introduction (right side of Figure 3A) were found in the bins corresponding to the range between 0.045 and 0.055 for *k_p_* and between 0.45 and 0.55 for *k_m_*. In contrast, the most frequent values estimated without the introduction (left side of Figure 3A) were found in the range between 0.065 and 0.075 for *k_p_*, while those for *k_m_* were correctly estimated. By using PSO (Figure 3B), *k_m_* was correctly estimated with the introduction, but *k_p_* failed, while the estimations of both parameters failed without the introduction. Furthermore, another difference between the estimations with and without the introduction is the distribution form of the estimated values, although the numbers of trial successes in the optimization were different with and without the introduction (see details in Methods). As seen in Figure 3A and 3A, the values with the introduction were sharply distributed, while those without the introduction were widely distributed. The introduction of DE constraints contracted the parameter space to facilitate the estimation of the correct values. As a result, the parameter accuracy was improved by the new objective function with the introduction of DE constraints in Model 1.

**Figure 4:**
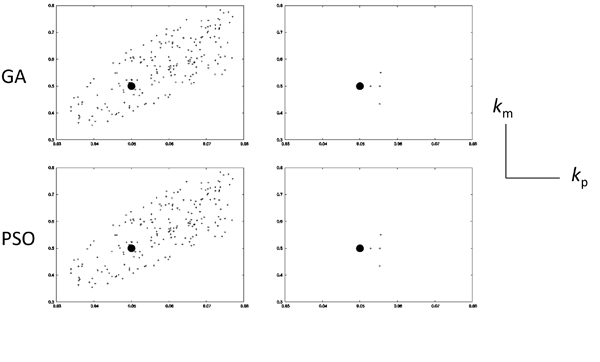
**Scatter plot of estimated parameter sets for Model 1** The distributions of the parameter sets by GA and PSO are shown with and without DE constraints (right and left sides, respectively). The black circles indicates the given parameter sets (*k_p_*=0.05 and *k_m_*=0.5).

Figure 4 clarifies the contraction of parameter space with the introduction of DE constraints into the objective function. Indeed, the estimated values with the introduction by using GA and PSO were concentrated around the correct values (right side of Figure 4). In contrast, the estimated values without the introduction by using the two optimization techniques were broadly distributed (left side). Although the numbers of estimated parameter sets were different with and without the introduction (see details in Methods), the distributions by using the two techniques without the introduction show weak positive correlations. This indicates that the ratio of estimated parameter sets was approximately kept, but the correct estimations failed, without the introduction. 

### Model 2

**Figure 5:**
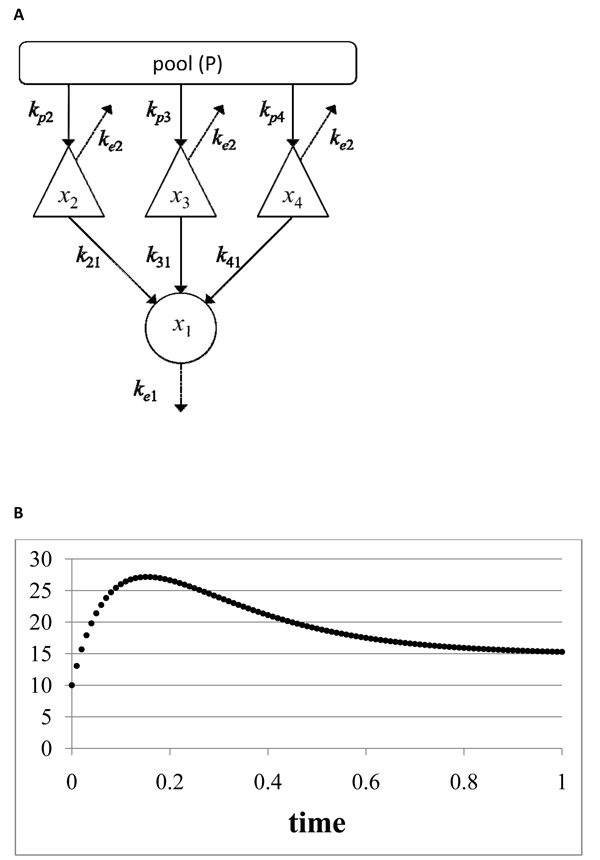
**Model 2: cascade reaction** The molecular cascade reaction of four molecules is schematically shown (A). According to the kinetics in the model (see details in Methods), a reference curve of one variable, *x*_1_, was generated for 0≦*t*≦1 with intervals of 0.01, under the following conditions: *x*_1_(0) = 10.0, *x*_2_(0) = 130.0, *x*_3_(0) =80.0, *x*_4_(0)=170.0, *k*_21_=5.0, *k*_31_=7.0, *k*_41_=11.0, *k_p_*_2_ = 3.0, *k_p_*_3_=4.0, *k_p_*_4_=10.0, *k_e_*_1_=5.0 and *k_e_*_2_=3.0 (B).

We analyzed a network model for the molecular cascade reaction of four molecules (Figure 5A). According to the kinetics of the model (see also Methods), the reference curve of one variable, *x*_1_, was generated (Figure 5B), and GA and PSO were applied to it to evaluate the effect of the introduction of DE constraints (see details in Additional File 2) into the objective function. 

**Figure 6:**
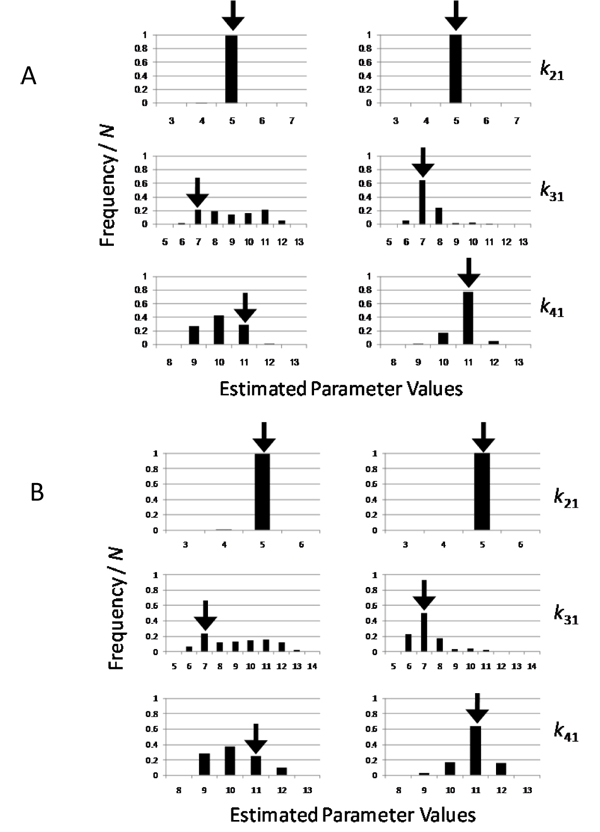
**Estimated parameter sets for Model 2** The parameter sets are estimated by using GA (A) and PSO (B), and in each figure, the histograms of parameter sets with and without DE constraints (right and left sides, respectively) are shown. The bin of the histogram indicates the fraction of the number of parameters within a range (1.0 for all parameters) to the total number of trial successes (200 for all cases) (see details in Methods).

In Model 2, the introduction of DE constraints into the objective function was also highly effective for correctly estimating the parameter values in both GA and PSO (Figure 6). By using GA (Figure 6A), all three parameters were correctly estimated with the introduction (right side), while the estimations of *k*_31_ and *k*_41_ failed without the introduction (left side). By using PSO (Figure 6B), all three parameters were also correctly estimated with the introduction (right side), while the estimations of *k*_41_ failed without the introduction (left side). Furthermore, the features of the distribution forms of the estimated values were similar to those in Model 1 (Figure 3). As seen in Figure 6A and 6B, the distribution of the estimated values with the introduction was sharp (right side), while that without the introduction was wide (left side). As a result, the parameter accuracy was also improved by the new objective function to contract the parameter space with the introduction of DE constraints in Model 2.

**Figure 7:**
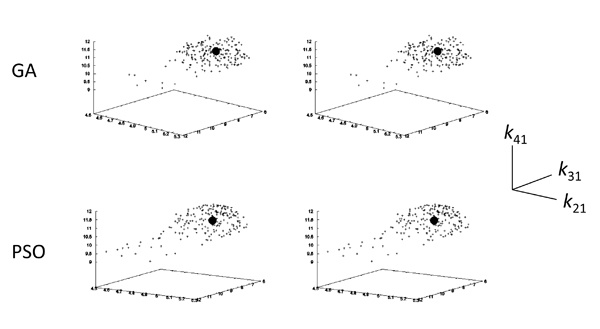
**Scatter plot of estimated parameter sets for Model 2** The distributions of the parameter sets by GA and PSO are shown with and without DE constraints (right and left sides, respectively). The black circles indicate the given parameter sets (*k*_21_=5.0, *k*_31_=7.0, and *k*_41_=11.0).

The contraction of parameter space with the introduction of DE constraints into the objective function is shown more clearly in Figure 7. The features of the parameter space in Figure 7 are similar to those in Figure 4. Indeed, the estimated values with the introduction by using GA and PSO were concentrated around the correct values (right side of Figure 7), while the estimated values without the introduction were broadly distributed (left side). In addition, the distributions by using the two techniques without the introduction also show weak positive correlations, similar to the case in Figure 4. Without the introduction, the ratio of estimated parameter sets was approximately maintained, but the correct estimations failed. 

## Discussion	

The introduction of DE constraints into the objective function clearly improved the parameter accuracy. Indeed, the parameter value sets were correctly estimated by the introduction of DE constraint into the objective function, while they were falsely estimated without the introduction. Furthermore, the parameter sets with the introduction were sharply distributed near the correct values in all cases, in contrast to the wide distribution without the introduction. In general, the derivatives included the information on the curve form of the measured time-series data, such as slope, extremal point and inflection point. This indicates that the new objective function estimates the difference of not only the values but also the forms between the measured and estimated data, while the standard objective function estimates only the value difference. Note that the DE constraint is rationally reduced from the original system of differential equations for a given model in a mathematical sense. Thus, our approach is expected to be a general approach in parameter optimization for improving the parameter accuracy. 

**Figure 8:**
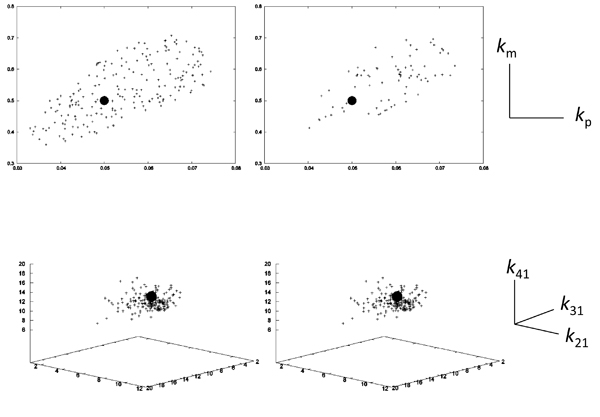
**Scatter plot of estimated parameter sets for the simulation data with noise for Models 1 and 2** The distributions of the parameter sets by GA for the data with noise (see details in Methods and Additional file 1) are shown for Model 1 (A) and Model 2 (B), with and without DE constraints (right and left sides, respectively). In this estimation, only two values under the optimization condition were slightly modified, in comparison with the case of data generation without noise: *α* was set to 0.995 in Model 1, and *E*/*T* was set to 0.05 in Model 2. The black circles indicate the given parameter sets for the two models.

To further test the performance of the present constraints in more realistic situations, we estimated the same parameters sets in Models 1 and 2 in the case of the simulated data with noise (see Methods). The reference curves for Models 1 and 2 were generated (Additional file 1), and the parameter sets were estimated by using GA with the same procedure as the case of the data without noise (Fig. 8). In both Models 1 and 2, the new constraints were also effective to improve the accuracy of parameter estimations. As the same as in Figures 4 and 7, the estimated values with the introduction were concentrated around the correct values (right side of Figure 8), while the estimated values without the introduction were broadly distributed (left side). However, the distribution ranges of parameter values in both models were widened in the data with noise, in comparison with those in the data without noise. Thus, our method may be more effective to the data curve obtained by some pre-processing methods than intact data, in the application of the present method to real data including noise. 

As expected, the new objective function requires more computational time, in comparison with an objective function with only a standard error function, due to the increase of the functions in DE constraints. Indeed, the computational time of our method was larger than that of the standard method in Models 1 and 2; the computational times for the standard method and our method were 0.4 and 2.3 hours in Model 1, and 0.03 and 0.22 hours in Model 2 (32 CPU’s of Intel(R) Xeon(R) X5550 2.67GHz). In addition to the computational time, a pitfall of our method is the equation size of DE constraints. In the equivalent systems, the number of terms frequently increases (see Additional file 3), and this may result in the difficulty of the application of our method to a complex or large model. Although we do not still reach a clear conclusion to overcome the difficulty, two ways can be considered. One way is an approximation method and the other is a mathematical manipulation method. As for the former method, in the DE constraints, the terms with a higher order of derivatives in the differential equations appeared frequently in the equivalent system (see Additional files 2 and 3). The magnitude of the estimated values of the higher order derivatives was relatively smaller than those of the lower order derivatives. If the estimation of terms with higher order derivatives can be neglected, then the computational time will be reduced. As for the latter method, we can use some equation-simplification methods by symbolic computation (personal communication from Drs. A. Sedoglavic, F. Lemaire and F. Boulier of Lille University). Indeed, the size of DE constraints for the negative feedback model with oscillation was reduced from 7.4MB obtained by the pure differential elimination in present procedure to 0.1MB after the equation simplification by symbolic computation (data not shown).  Further studies will be needed to shorten the computational time by the combination of the approximation and the simplification of the DE constraints.

**Figure 9:**
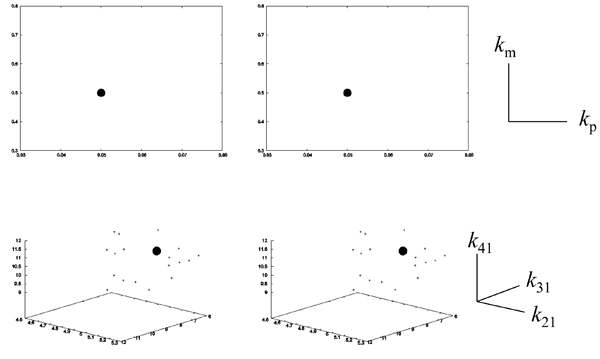
**Scatter plot of estimated parameter sets by the modified Powell method for Models 1 and 2** The distributions of the parameter sets by the modified Powell method are shown for Model 1 (upper) and Model 2 (lower), with and without DE constraints (right and left sides, respectively). The black circles indicate the given parameter sets for the two models.

Furthermore, more local minima in the objective function appeared by introducing the DE constraints, also due to the increase in the functions. Indeed, the number of successful estimations by GA in our method was less than that of the standard method in Model 1. To further survey the effects of the landscape of DE constraints on the parameter estimation, we performed parameter optimization by using a gradient method, the modified Powell method [8,9]. While the evolutionary optimization techniques, such as GA and PSO, equip the algorithm to jump from the trap of local minima, the gradient method generally stop to estimate the parameter values in the valley of the local minima. The parameter values for the two models obtained by using the objective functions with and without the DE constraints are shown in Fig. 9. In Model 2, the situation was similar to the case where the evolutionary techniques were adopted in Figs. 6 and 7. Indeed, the parameter space was clearly contracted under the influence of the introduction of DE constraints. In contrast, in Model 1, our method failed to estimate the parameter values, due to the lack of an error function below a given threshold, while the standard method succeeded with the broad parameter space. This indicates a pitfall, in that the risk of being trapped by local minima increases in the objective function with DE constraints, in comparison with the risk in the objective function without DE constraints. Thus, the introduction of DE constraints into the objective function is more suitable for the evolutionary optimization techniques than the gradient based techniques.

One possible use of our method is its application to network inference without known structure. Since the present method is designed with the assumption of a known network structure, the application range of our method to network inference is naturally restricted. However, our method can select the most possible network structure among the networks with similar structures. Indeed, we designed a similar procedure for evaluating the network structures with measured data [10]. In our previous approach, we adopted the transformation of a system of differential equations into the equivalent system of algebraic equations by Laplace transformation. In this case, the system must be linear, due to the Laplace transformation. Furthermore, the numeric optimization in the previous approach frequently faces difficulties, due to the existence of the pole in the Laplace domain. In contrast, these pitfalls are overcome in the present method, by introducing the constraints by differential elimination. This supports the application of the present method to the model selection issue.

Various models for describing biological phenomena are available [11]. In particular, several feedback models are important for describing the biological phenomena [12,13]. Although the performance of our approach for the two representative models in biological phenomena was tested in this study, further tests for the performance of the DE constraint introduction remain for the models that are important in systems and synthetic biology. In the near future, we will report the evaluation of our approach in the cases of various models, in addition to the reduction of computational time and the trials of model selection. 

## Conclusions 

The introduction of the constraints by using differential elimination was effectively improved the parameter accuracy in two models of linear and nonlinear equations, especially when we assumed that unmeasured variables were included, by two optimization techniques. This clearly indicates that the ability of our method for estimating the parameter values was far superior to that of various methods with the standard error function. Although the present study focused on two simple models, our method is a feasible approach for parameter estimation in network dynamics. 

## Methods

### Analyzed models

The system of differential equations in Model 1 is expressed as follows:

								(4)

We assume that the model expresses the binding and dissociation between two molecules, and that only one complex, *x_AB_*, can be measured.

The system of differential equations in Model 2 is expressed as follows:

							(5)

We assume that the molecules, *x*_2_, *x*_3_, and *x*_4_, activate *x*_1_ with linear relationships, and that only one molecule, *x*_1_, can be measured.

The data with noise were generated by Box-Muller method [14]. Each of data, *X*_e_(*t*), is expressed as follows:

,

where *X*(*t*) is a value at time *t* in original curve of Figures 2 and 5, *Rn* is random variable according to the standard normal distribution, and *c* was set to 0.666. 

### Optimization techniques

Two well-known parameter optimization techniques, the genetic algorithm (GA) [15-19] and the particle swarm optimization (PSO) [20,21], were used. In the parameter optimization, two thresholds were set to stop the optimization: the average value of the error function over time points, *E*/*T*, and the number of generations per optimization. In this study, we performed the optimization 200 times in both techniques, and the thresholds of *E*/*T* were set to 0.01 for Model 1 and 0.001 for Model 2, and the threshold for generation number was set to 2000. As a result, the numbers of successes by 200 trials were 200 without DE constraints and 51 by GA and 11 by PSO with DE constraints, for Model 1, and 200 for all cases for Model 2. 

### Introduction of the new constraints into the objective function 

The objective function in this study is composed of two terms: one is the standard error function between the estimated and measured data, and the other is the constraints obtained by differential elimination. The error function is defined as follows: Suppose that *x^c^_i,t_* is the time-course data at time *t* of *x_i_* calculated by using the estimated parameter values, and *x^m^_i,t_* represents the measured data at time  *t*. The sum of the absolute value of the relative error between *x^c^_i,t_* and *x^m^_i,t_* gives the total relative error, *E*, as a standard error function, i.e.,

,									(6)

where *N* and *T* are the number of variables and the time points, respectively: *N* was 2 for Model 1 and 4 for Model 2, and *T* was 100.

Next we define the constraints obtained by differential elimination.  In general, differential elimination rewrites the original system of differential equations into an equivalent system, which means that the number of equations is equal in both systems. Thus, we can express the constraint by differential elimination, *C*_DE_, as the linear combination of the equations in the equivalent system, as follows:

,									(7)

where *L* and *T* are the numbers of equivalent equations and time points, respectively: *L* was 2 for Model 1 and 5 for Model 2.

Finally, we introduce *C*_DE_ into the objective function, *OF*, in combination with *E*, as:

									(8)

where *α* the a weight of two functions, which is approximately estimated by a Pareto optimal solutions for *E* and *C* and then is manually modified. In the present study, *α* was set to 0.1 in Model 1 and 0.9999999 in Model 2. As a result, our computational task is to determine a set of parameter values that minimize to *OF*.

### Implementation of differential elimination

All of the symbolic computations for the differential elimination were performed using the *diffalg* package of MAPLE 10. In the performance of differential elimination, the ranking of variables was: *x*_A_ ≻ *x*_B_ ≻ *x*_AB_ in Model 1 and P(Pool) ≻ *x*_4_ ≻ *x*_3_ ≻ *x*_2_ ≻ *x*_1_ in Model 2. Subsequently, we converted the form of the polynomial equations derived by differential elimination to the Java code by using the *CodeGeneration* feature in Maple 10.

## Competing interests

The authors declare that they have no competing interests.

## Authors' contributions

MN performed the implementation and the calculations, and participated in the design of the study. KH conceived of the study, participated in its design and coordination, and drafted the manuscript. MO, YT, and JM participated in the design of the study, and helped to draft the manuscript. All authors read and approved of the final manuscript.

## Supplementary Material

Additional file 1 According to the kinetics of the models for Models 1 and 2, the reference data of one variable, *x*_AB_ (A), and that of one variable, *x*_1_ (B), were generated under the same conditions as those in Figures 2 and 5. Click here for file

Additional file 2 The equivalent equations for Model 1 were derived from the system of differential equations by differential elimination. Click here for file

Additional file 3 The equivalent equations for Model 2 were derived from the system of differential equations by differential elimination. Click here for file
