# Supplementary figures and images for: Parameter optimization by using differential elimination: a general approach for introducing constraints into objective functions
Source: BMC Syst Biol. 2010 Sep 13;4(Suppl 2):S9. doi: 10.1186/1752-0509-4-S2-S9 (PMC2982696; doi:10.1186/1752-0509-4-S2-S9)

Model 1

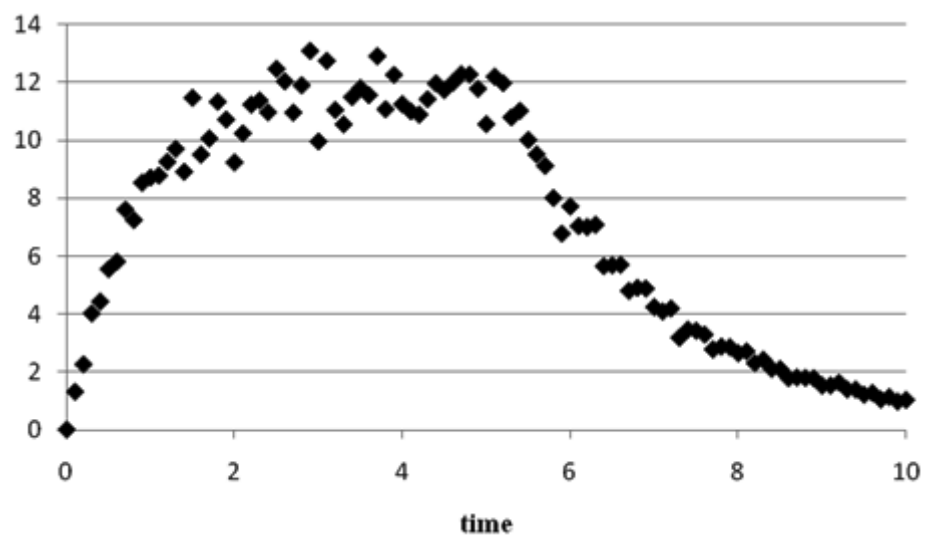

Model 2

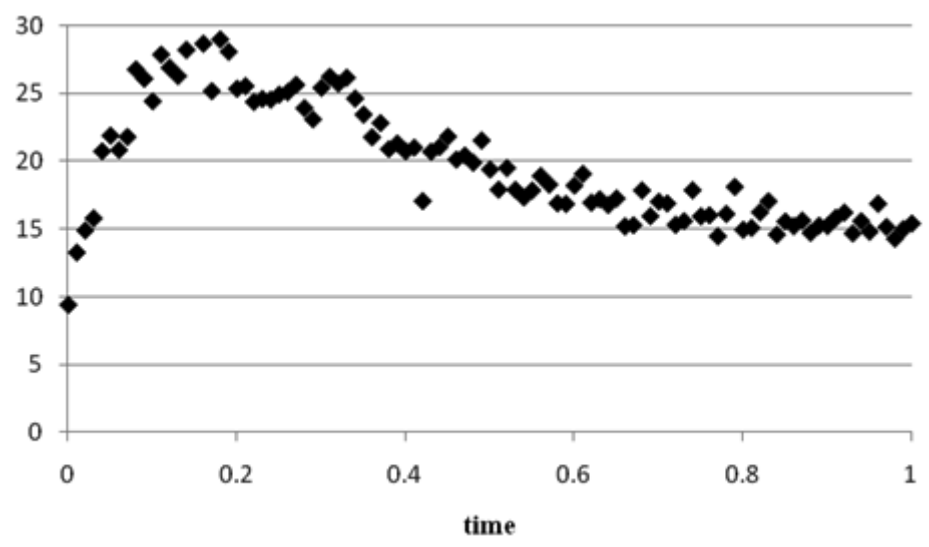

Supplement: Additional file 1 — According to the kinetics of the models for Models 1 and 2, the reference data of one variable, xAB (A), and that of one variable, x1 (B), were generated under the same conditions as those in Figures 2 and 5. [file 1752-0509-4-S2-S9-S1.pdf]
