# Supplementary material for: Parameter optimization by using differential elimination: a general approach for introducing constraints into objective functions
Source: BMC Syst Biol. 2010 Sep 13;4(Suppl 2):S9. doi: 10.1186/1752-0509-4-S2-S9 (PMC2982696; doi:10.1186/1752-0509-4-S2-S9)
Supplement: Additional file 2 — The equivalent equations for Model 1 were derived from the system of differential equations by differential elimination. [file 1752-0509-4-S2-S9-S2.pdf]

$$C_{1,t} = \frac{\frac{d}{dt} x_{AB}\left(t\right) + k_{m}\, x_{AB}\left(t\right)}{k_{p}\, x_{A}} - x_{B}\left(t\right) = 0 \tag{1}$$

$$C_{2,t} = -\left(\frac{d}{dt} x_{AB}\left(t\right)\right)\left(k_{m} + k_{p}\, x_{A}\right) - \frac{d^{2}}{dt^{2}} x_{AB}\left(t\right) = 0 \tag{2}$$
