# Supplementary material for: Parameter optimization by using differential elimination: a general approach for introducing constraints into objective functions
Source: BMC Syst Biol. 2010 Sep 13;4(Suppl 2):S9. doi: 10.1186/1752-0509-4-S2-S9 (PMC2982696; doi:10.1186/1752-0509-4-S2-S9)
Supplement: Additional file 3 — The equivalent equations for Model 2 were derived from the system of differential equations by differential elimination. [file 1752-0509-4-S2-S9-S3.pdf]

$$
\begin{aligned}
C_{1,t} = {} & (k_{21}\, k_{e2}\, (\frac{d}{dt}\, x_1(t))\, k_{41} + k_{21}\, k_{41}\, (\frac{d}{dt}\, x_1(t))\, k_{31} \\
& + k_{21}\, k_{e2}\, (\frac{d}{dt}\, x_1(t))\, k_{31} + k_{21}\, k_{41}\, k_{e1}\, (\frac{d}{dt}\, x_1(t)) + k_{21}\, k_{e1}\, (\frac{d}{dt}\, x_1(t))\, k_{31} \\
& + k_{21}\, k_{e2}^2\, k_{e1}\, x_1(t) + k_{e2}^2\, k_{e1}\, x_1(t)\, k_{31} + k_{41}\, k_{e1}\, (\frac{d}{dt}\, x_1(t))\, k_{31} \\
& + k_{41}\, (\frac{d}{dt}\, x_1(t))\, k_{31}\, k_{e2} + k_{e2}^2\, k_{e1}\, x_1(t)\, k_{41} + (\frac{d}{dt}\, x_1(t))\, k_{e2}^3 \\
& + k_{21}\, k_{e2}^2\, (\frac{d}{dt}\, x_1(t)) + k_{e2}^2\, (\frac{d}{dt}\, x_1(t))\, k_{41} + k_{e2}^3\, k_{e1}\, x_1(t) \\
& + k_{21}\, k_{41}\, k_{e1}\, x_1(t)\, k_{31} + k_{41}\, k_{e2}\, k_{e1}\, x_1(t)\, k_{31} + k_{e2}^2\, (\frac{d}{dt}\, x_1(t))\, k_{31} \\
& + 2\, k_{21}\, k_{e2}\, k_{e1}\, (\frac{d}{dt}\, x_1(t)) + 2\, k_{41}\, k_{e2}\, k_{e1}\, (\frac{d}{dt}\, x_1(t)) \\
& + 2\, k_{e2}\, k_{e1}\, (\frac{d}{dt}\, x_1(t))\, k_{31} + k_{21}\, k_{41}\, k_{e2}\, k_{e1}\, x_1(t) \\
& + k_{21}\, k_{e2}\, k_{e1}\, x_1(t)\, k_{31} + 3\, k_{e2}^2\, k_{e1}\, (\frac{d}{dt}\, x_1(t)) + k_{e1}\, (\frac{d^3}{dt^3}\, x_1(t)) \\
& + k_{41}\, (\frac{d^3}{dt^3}\, x_1(t)) + (\frac{d^3}{dt^3}\, x_1(t))\, k_{31} + k_{21}\, (\frac{d^3}{dt^3}\, x_1(t)) + 3\, k_{e2}\, (\frac{d^3}{dt^3}\, x_1(t)) \\
& + 3\, k_{e2}^2\, (\frac{d^2}{dt^2}\, x_1(t)) + (\frac{d^4}{dt^4}\, x_1(t)) + k_{)}21\, k_{41}\, (\frac{d^2}{dt^2}\, x_1(t)) + k_{21}\, k_{e1}\, (\frac{d^2}{dt^2}\, x_1(t)) \\
& + k_{e1}\, (\frac{d^2}{dt^2}\, x_1(t))\, k_{31} + k_{41}\, (\frac{d^2}{dt^2}\, x_1(t))\, k_{31} + k_{41}\, k_{e1}\, (\frac{d^2}{dt^2}\, x_1(t)) \\
& + k_{21}\, (\frac{d^2}{dt^2}\, x_1(t))\, k_{31} + 3\, k_{e2}\, k_{e1}\, (\frac{d^2}{dt^2}\, x_1(t)) + 2\, k_{e2}\, (\frac{d^2}{dt^2}\, x_1(t))\, k_{41} \\
& + 2\, k_{e2}\, (\frac{d^2}{dt^2}\, x_1(t))\, k_{31} + 2\, k_{21}\, k_{e2}\, (\frac{d^2}{dt^2}\, x_1(t)))/(k_{21}\, k_{p2}\, k_{e2}^2 \\
& + k_{21}\, k_{31}\, k_{41}\, k_{p2} + k_{21}\, k_{41}\, k_{p2}\, k_{e2} + k_{21}\, k_{31}\, k_{p2}\, k_{e2} \\
& + k_{21}\, k_{41}\, k_{p3}\, k_{31} + k_{21}\, k_{p4}\, k_{e2}\, k_{41} + k_{21}\, k_{p3}\, k_{e2}\, k_{31} \\
& + k_{21}\, k_{p4}\, k_{41}\, k_{31} + k_{p3}\, k_{31}\, k_{e2}\, k_{41} + k_{p4}\, k_{41}\, k_{e2}\, k_{31} + k_{41}\, k_{e2}^2\, k_{p4} \\
& + k_{31}\, k_{e2}^2\, k_{p3}) - x_5(t) = 0
\end{aligned} \tag{1}
$$

$$
\begin{aligned}
C_{2,t} = {} & (k_{p4}\, k_{41}^3\, k_{e2}^2\, (\frac{d}{dt}\, x_1(t)) - k_{41}^2\, k_{e2}\, k_{e1}\, x_1(t)\, k_{31}^2\, k_{p4} \\
& - k_{21}\, k_{41}^2\, k_{e1}\, (\frac{d}{dt}\, x_1(t))\, k_{p4}\, k_{e2} - k_{21}\, k_{41}^2\, k_{e1}\, (\frac{d}{dt}\, x_1(t))\, k_{p4}\, k_{31} \\
& - k_{21}\, k_{e2}^2\, (\frac{d}{dt}\, x_1(t))\, k_{41}^2\, k_{p4} - k_{41}^2\, k_{e1}\, x_1(t)\, k_{31}\, k_{e2}^2\, k_{p4} \\
& + k_{21}\, k_{e2}\, (\frac{d}{dt}\, x_1(t))\, k_{31}^2\, k_{p4}\, k_{41} + k_{21}\, k_{e2}^2\, (\frac{d}{dt}\, x_1(t))\, k_{31}\, k_{p4}\, k_{41} \\
& - k_{21}\, k_{e2}\, k_{e1}\, (\frac{d}{dt}\, x_1(t))\, k_{p4}\, k_{41}\, k_{31} + k_{41}^2\, k_{e2}^2\, k_{e1}\, (\frac{d}{dt}\, x_1(t))\, k_{p4} \\
& - k_{41}\, k_{p4}\, k_{e2}^3\, k_{e1}\, (\frac{d}{dt}\, x_1(t)) - k_{21}^2\, k_{p4}\, k_{41}^2\, k_{e1}\, (\frac{d}{dt}\, x_1(t)) \\
& - k_{21}^2\, k_{p4}\, k_{41}\, k_{e2}\, k_{e1}\, (\frac{d}{dt}\, x_1(t)) - k_{21}^2\, k_{p2}\, k_{e2}^2\, k_{e1}\, (\frac{d}{dt}\, x_1(t))
\end{aligned} \tag{2}
$$

$$-k_{21}^2\,k_{31}^2\,k_{p2}\,k_{e1}\,(\frac{d}{dt}\,x_1(t)) + k_{p4}\,k_{41}^3\,k_{e2}\,k_{e1}\,x_1(t)\,k_{31}$$
$$+k_{p4}\,k_{41}^3\,k_{e2}\,(\frac{d}{dt}\,x_1(t))\,k_{31} + k_{p4}\,k_{41}^3\,k_{e1}\,(\frac{d}{dt}\,x_1(t))\,k_{31}$$
$$-k_{21}^2\,k_{p3}\,k_{31}^2\,k_{e1}\,(\frac{d}{dt}\,x_1(t)) - k_{21}^2\,k_{p3}\,k_{31}\,k_{e2}\,k_{e1}\,(\frac{d}{dt}\,x_1(t))$$
$$-k_{31}\,k_{41}^2\,k_{e1}\,(\frac{d}{dt}\,x_1(t))\,k_{p4}\,k_{e2} - k_{31}\,k_{p3}\,k_{e2}^3\,k_{e1}\,(\frac{d}{dt}\,x_1(t))$$
$$-k_{31}^2\,k_{p4}\,k_{41}^2\,k_{e1}\,(\frac{d}{dt}\,x_1(t)) - k_{31}^2\,k_{p4}\,k_{41}\,k_{e2}\,k_{e1}\,(\frac{d}{dt}\,x_1(t))$$
$$-k_{31}^2\,k_{e2}^2\,k_{e1}\,(\frac{d}{dt}\,x_1(t))\,k_{p3} - k_{21}\,k_{41}^2\,(\frac{d}{dt}\,x_1(t))\,k_{31}^2\,k_{p4}$$
$$+k_{21}\,k_{e2}^2\,k_{e1}\,x_1(t)\,k_{p4}\,k_{41}\,k_{31} + k_{21}^2\,k_{e1}\,x_1(t)\,k_{31}^2\,k_{p4}\,k_{41}$$
$$-k_{21}\,k_{31}^2\,k_{e2}\,k_{p2}\,k_{e1}\,(\frac{d}{dt}\,x_1(t)) - k_{21}\,k_{p2}\,k_{e2}^3\,k_{e1}\,(\frac{d}{dt}\,x_1(t))$$
$$-k_{41}^2\,(\frac{d}{dt}\,x_1(t))\,k_{31}\,k_{e2}^2\,k_{p4} + k_{21}\,k_{e2}\,k_{e1}\,x_1(t)\,k_{31}^2\,k_{p4}\,k_{41}$$
$$+k_{21}^2\,(\frac{d}{dt}\,x_1(t))\,k_{31}^2\,k_{p4}\,k_{41} - k_{21}\,k_{41}^2\,k_{e2}^2\,k_{e1}\,x_1(t)\,k_{p4}$$
$$+k_{p4}\,k_{41}^3\,k_{e2}^2\,k_{e1}\,x_1(t) - k_{21}\,k_{41}^2\,k_{e1}\,x_1(t)\,k_{31}^2\,k_{p4}$$
$$+k_{21}\,k_{p4}\,k_{41}^3\,(\frac{d}{dt}\,x_1(t))\,k_{31} + k_{21}\,k_{p4}\,k_{41}^3\,k_{e2}\,(\frac{d}{dt}\,x_1(t))$$
$$+k_{21}\,k_{p4}\,k_{41}^3\,k_{e1}\,(\frac{d}{dt}\,x_1(t)) - k_{21}^2\,k_{p4}\,k_{41}^2\,k_{e2}\,k_{e1}\,x_1(t)$$
$$+k_{21}^2\,k_{p4}\,k_{41}\,k_{e2}\,(\frac{d}{dt}\,x_1(t))\,k_{31} - k_{21}^2\,k_{p4}\,k_{41}^2\,k_{e1}\,x_1(t)\,k_{31}$$
$$+k_{21}^2\,k_{p4}\,k_{41}\,k_{e2}\,k_{e1}\,x_1(t)\,k_{31} - k_{21}^2\,k_{p4}\,k_{41}^2\,(\frac{d}{dt}\,x_1(t))\,k_{31}$$
$$-k_{21}^2\,k_{p4}\,k_{41}^2\,k_{e2}\,(\frac{d}{dt}\,x_1(t)) - k_{e2}\,(\frac{d}{dt}\,x_1(t))\,k_{41}^2\,k_{31}^2\,k_{p4}$$
$$+k_{21}\,k_{p4}\,k_{41}^3\,k_{e1}\,x_1(t)\,k_{31} + k_{21}\,k_{p4}\,k_{41}^3\,k_{e2}\,k_{e1}\,x_1(t)$$
$$+k_{41}^2\,k_{e2}^2\,(\frac{d^2}{dt^2}\,x_1(t))\,k_{p4} - k_{41}\,k_{p4}\,k_{e2}^3\,(\frac{d^2}{dt^2}\,x_1(t))$$
$$+k_{p4}\,k_{41}^3\,k_{e1}\,(\frac{d^2}{dt^2}\,x_1(t)) + k_{p4}\,k_{41}^2\,k_{e1}\,(\frac{d^3}{dt^3}\,x_1(t))$$
$$+k_{p4}\,k_{41}^3\,(\frac{d^2}{dt^2}\,x_1(t))\,k_{31} - k_{41}\,(\frac{d^4}{dt^4}\,x_1(t))\,k_{p4}\,k_{e2}$$
$$-k_{21}^2\,k_{31}^2\,k_{p2}\,(\frac{d^2}{dt^2}\,x_1(t)) - k_{21}^2\,k_{p2}\,k_{e2}\,(\frac{d^3}{dt^3}\,x_1(t))$$
$$-k_{21}^2\,k_{p2}\,k_{e2}^2\,(\frac{d^2}{dt^2}\,x_1(t)) - k_{21}\,k_{31}\,k_{p2}\,(\frac{d^4}{dt^4}\,x_1(t))$$
$$-k_{21}\,k_{31}^2\,k_{p2}\,(\frac{d^3}{dt^3}\,x_1(t)) - k_{21}\,k_{p2}\,(\frac{d^4}{dt^4}\,x_1(t))\,k_{e2}$$
$$-k_{21}\,k_{p2}\,k_{e2}^3\,(\frac{d^2}{dt^2}\,x_1(t)) - k_{31}\,k_{41}\,k_{p4}\,(\frac{d^4}{dt^4}\,x_1(t))$$
$$-k_{31}\,k_{p3}\,k_{e2}^3\,(\frac{d^2}{dt^2}\,x_1(t)) - k_{31}\,(\frac{d^4}{dt^4}\,x_1(t))\,k_{p3}\,k_{e2}$$
$$-k_{31}^2\,(\frac{d^3}{dt^3}\,x_1(t))\,k_{p3}\,k_{e2} - k_{31}^2\,k_{e2}^2\,(\frac{d^2}{dt^2}\,x_1(t))\,k_{p3}$$

$$-k_{31}^2\,k_{p4}\,k_{41}^2\,(\frac{d^2}{dt^2}\,x_1(t)) - k_{31}^2\,k_{p4}\,k_{41}\,(\frac{d^3}{dt^3}\,x_1(t))$$
$$-k_{21}^2\,k_{p4}\,k_{41}^2\,(\frac{d^2}{dt^2}\,x_1(t)) - k_{21}^2\,k_{p4}\,k_{41}\,(\frac{d^3}{dt^3}\,x_1(t))$$
$$-k_{21}^2\,k_{p3}\,k_{31}\,(\frac{d^3}{dt^3}\,x_1(t)) - k_{21}^2\,k_{p3}\,k_{31}^2\,(\frac{d^2}{dt^2}\,x_1(t))$$
$$-k_{21}\,k_{41}\,k_{p4}\,(\frac{d^4}{dt^4}\,x_1(t)) - k_{21}\,k_{p3}\,k_{31}\,(\frac{d^4}{dt^4}\,x_1(t))$$
$$-k_{21}^2\,k_{p2}\,(\frac{d^3}{dt^3}\,x_1(t))\,k_{31} + k_{21}\,k_{p4}\,k_{41}^3\,(\frac{d^2}{dt^2}\,x_1(t))$$
$$-k_{21}\,k_{p3}\,k_{31}^2\,(\frac{d^3}{dt^3}\,x_1(t)) + 2\,k_{41}^3\,(\frac{d^2}{dt^2}\,x_1(t))\,k_{p4}\,k_{e2}$$
$$+2\,k_{41}^2\,(\frac{d^3}{dt^3}\,x_1(t))\,k_{p4}\,k_{e2} - 2\,k_{41}\,k_{p4}\,k_{e2}^2\,(\frac{d^3}{dt^3}\,x_1(t))$$
$$-2\,k_{21}\,k_{p2}\,k_{e2}^2\,(\frac{d^3}{dt^3}\,x_1(t)) - 2\,k_{31}\,k_{p3}\,k_{e2}^2\,(\frac{d^3}{dt^3}\,x_1(t))$$
$$-2\,k_{21}\,k_{41}^2\,k_{e1}\,x_1(t)\,k_{31}\,k_{p4}\,k_{e2} - 2\,k_{21}\,k_{e2}^2\,k_{e1}\,(\frac{d}{dt}\,x_1(t))\,k_{p4}\,k_{41}$$
$$-2\,k_{21}\,k_{e2}^2\,k_{e1}\,(\frac{d}{dt}\,x_1(t))\,k_{p3}\,k_{31} - 2\,k_{21}^2\,k_{31}\,k_{e2}\,k_{p2}\,k_{e1}\,(\frac{d}{dt}\,x_1(t))$$
$$+2\,k_{41}^3\,k_{e1}\,(\frac{d}{dt}\,x_1(t))\,k_{p4}\,k_{e2} - 2\,k_{31}\,k_{41}\,k_{e2}^2\,k_{e1}\,(\frac{d}{dt}\,x_1(t))\,k_{p4}$$
$$-2\,k_{21}\,k_{e1}\,(\frac{d}{dt}\,x_1(t))\,k_{31}^2\,k_{p3}\,k_{e2} - 2\,k_{21}\,k_{41}^2\,(\frac{d}{dt}\,x_1(t))\,k_{31}\,k_{p4}\,k_{e2}$$
$$-2\,k_{21}\,k_{31}\,k_{p2}\,k_{e2}^2\,k_{e1}\,(\frac{d}{dt}\,x_1(t)) - k_{21}\,k_{e1}\,(\frac{d^2}{dt^2}\,x_1(t))\,k_{p4}\,k_{41}\,k_{31}$$
$$-k_{21}\,k_{41}^2\,(\frac{d^2}{dt^2}\,x_1(t))\,k_{p4}\,k_{31} - k_{21}\,(\frac{d^3}{dt^3}\,x_1(t))\,k_{p4}\,k_{41}\,k_{31}$$
$$-k_{21}^2\,k_{p4}\,k_{41}\,k_{e1}\,(\frac{d^2}{dt^2}\,x_1(t)) - k_{21}^2\,k_{p4}\,k_{41}\,k_{e2}\,(\frac{d^2}{dt^2}\,x_1(t))$$
$$-k_{21}^2\,k_{p2}\,k_{e2}\,k_{e1}\,(\frac{d^2}{dt^2}\,x_1(t)) + k_{p4}\,k_{41}^3\,(\frac{d^3}{dt^3}\,x_1(t)) + k_{p4}\,k_{41}^2\,(\frac{d^4}{dt^4}\,x_1(t))$$
$$-k_{21}\,k_{p2}\,k_{e1}\,(\frac{d^3}{dt^3}\,x_1(t))\,k_{e2} - k_{21}\,k_{31}^2\,k_{p2}\,k_{e1}\,(\frac{d^2}{dt^2}\,x_1(t))$$
$$-k_{21}\,k_{31}^2\,k_{e2}\,k_{p2}\,(\frac{d^2}{dt^2}\,x_1(t)) - k_{21}^2\,k_{p3}\,k_{31}\,k_{e2}\,(\frac{d^2}{dt^2}\,x_1(t))$$
$$-k_{21}^2\,k_{p3}\,k_{31}\,k_{e1}\,(\frac{d^2}{dt^2}\,x_1(t)) - k_{21}\,k_{41}\,k_{p4}\,k_{e1}\,(\frac{d^3}{dt^3}\,x_1(t))$$
$$-k_{21}\,k_{41}^2\,(\frac{d^2}{dt^2}\,x_1(t))\,k_{p4}\,k_{e2} - k_{31}\,k_{41}^2\,(\frac{d^2}{dt^2}\,x_1(t))\,k_{p4}\,k_{e2}$$
$$-k_{31}\,k_{41}\,k_{p4}\,k_{e1}\,(\frac{d^3}{dt^3}\,x_1(t)) - k_{31}\,k_{e1}\,(\frac{d^3}{dt^3}\,x_1(t))\,k_{p3}\,k_{e2}$$
$$-k_{31}^2\,k_{p4}\,k_{41}\,k_{e1}\,(\frac{d^2}{dt^2}\,x_1(t)) - k_{31}^2\,k_{p4}\,k_{41}\,k_{e2}\,(\frac{d^2}{dt^2}\,x_1(t))$$
$$-k_{31}^2\,k_{e1}\,(\frac{d^2}{dt^2}\,x_1(t))\,k_{p3}\,k_{e2} - k_{41}\,k_{e1}\,(\frac{d^3}{dt^3}\,x_1(t))\,k_{p4}\,k_{e2}$$
$$-k_{21}\,k_{31}\,k_{p2}\,k_{e1}\,(\frac{d^3}{dt^3}\,x_1(t)) - k_{21}\,k_{e2}\,(\frac{d^2}{dt^2}\,x_1(t))\,k_{p4}\,k_{41}\,k_{31}$$
$$-k_{21}\,k_{p3}\,k_{31}\,k_{e1}\,(\frac{d^3}{dt^3}\,x_1(t)) - k_{21}^2\,k_{p2}\,k_{e1}\,(\frac{d^2}{dt^2}\,x_1(t))\,k_{31}$$

$$-k_{21}\,k_{p3}\,k_{31}^2\,k_{e1}\,(\frac{d^2}{dt^2}\,x_1(t)) - 3\,k_{21}\,(\frac{d^3}{dt^3}\,x_1(t))\,k_{p4}\,k_{e2}\,k_{41}$$
$$-3\,k_{21}\,(\frac{d^3}{dt^3}\,x_1(t))\,k_{p3}\,k_{e2}\,k_{31} - 2\,k_{21}\,k_{e2}^2\,(\frac{d^2}{dt^2}\,x_1(t))\,k_{p3}\,k_{31}$$
$$-2\,k_{21}\,k_{e2}^2\,(\frac{d^2}{dt^2}\,x_1(t))\,k_{p4}\,k_{41} - 3\,k_{21}\,k_{e1}\,(\frac{d^2}{dt^2}\,x_1(t))\,k_{p4}\,k_{e2}\,k_{41}$$
$$-3\,k_{21}\,k_{e1}\,(\frac{d^2}{dt^2}\,x_1(t))\,k_{p3}\,k_{e2}\,k_{31} - 2\,k_{21}^2\,k_{31}\,k_{e2}\,k_{p2}\,(\frac{d^2}{dt^2}\,x_1(t))$$
$$-2\,k_{21}\,k_{p2}\,k_{e2}^2\,k_{e1}\,(\frac{d^2}{dt^2}\,x_1(t)) - 2\,k_{21}\,k_{31}\,k_{p2}\,k_{e2}^2\,(\frac{d^2}{dt^2}\,x_1(t))$$
$$-3\,k_{21}\,k_{31}\,k_{p2}\,k_{e2}\,(\frac{d^3}{dt^3}\,x_1(t)) - 2\,k_{31}\,k_{41}\,k_{e2}^2\,(\frac{d^2}{dt^2}\,x_1(t))\,k_{p4}$$
$$-3\,k_{31}\,k_{41}\,(\frac{d^3}{dt^3}\,x_1(t))\,k_{p4}\,k_{e2} - 3\,k_{31}\,k_{41}\,k_{e1}\,(\frac{d^2}{dt^2}\,x_1(t))\,k_{p4}\,k_{e2}$$
$$-2\,k_{31}\,k_{p3}\,k_{e2}^2\,k_{e1}\,(\frac{d^2}{dt^2}\,x_1(t)) - 2\,k_{21}\,(\frac{d^2}{dt^2}\,x_1(t))\,k_{31}^2\,k_{p3}\,k_{e2}$$
$$+2\,k_{41}^2\,k_{e1}\,(\frac{d^2}{dt^2}\,x_1(t))\,k_{p4}\,k_{e2} - 2\,k_{41}\,k_{p4}\,k_{e2}^2\,k_{e1}\,(\frac{d^2}{dt^2}\,x_1(t))$$
$$-3\,k_{21}\,k_{31}\,k_{p2}\,k_{e2}\,k_{e1}\,(\frac{d^2}{dt^2}\,x_1(t)))/(k_{41}(k_{p3}\,k_{31}\,k_{41}^3\,k_{e2}$$
$$+k_{41}^2\,k_{p3}\,k_{31}\,k_{e2}^2 + k_{21}\,k_{41}^3\,k_{p3}\,k_{31} + k_{21}\,k_{p2}\,k_{41}^3\,k_{e2}$$
$$+k_{21}\,k_{p2}\,k_{41}^3\,k_{31} + k_{21}\,k_{p2}\,k_{41}^2\,k_{e2}^2 - k_{31}^2\,k_{41}^2\,k_{p4}\,k_{e2}$$
$$-k_{21}^2\,k_{41}\,k_{p2}\,k_{e2}^2 + k_{21}^2\,k_{31}\,k_{p2}\,k_{e2}^2 - k_{21}^2\,k_{41}^2\,k_{p2}\,k_{e2}$$
$$+k_{21}^2\,k_{31}^2\,k_{41}\,k_{p2} - k_{21}^2\,k_{31}\,k_{41}^2\,k_{p2} + k_{21}^2\,k_{31}^2\,k_{p2}\,k_{e2}$$
$$-k_{21}\,k_{31}^2\,k_{41}^2\,k_{p2} + k_{31}\,k_{p4}\,k_{41}^3\,k_{e2} - k_{31}^2\,k_{41}\,k_{e2}^2\,k_{p3}$$
$$-k_{31}^2\,k_{41}^2\,k_{e2}\,k_{p3} - k_{21}\,k_{41}^2\,k_{31}^2\,k_{p4} + k_{21}\,k_{e2}^2\,k_{p3}\,k_{31}^2$$
$$+k_{21}\,k_{p4}\,k_{41}^3\,k_{31} + k_{21}\,k_{p4}\,k_{41}^3\,k_{e2} - k_{31}\,k_{41}^2\,k_{e2}^2\,k_{p4}$$
$$-k_{21}\,k_{41}^2\,k_{31}^2\,k_{p3} - k_{21}^2\,k_{41}^2\,k_{p3}\,k_{31} - k_{21}\,k_{41}^2\,k_{e2}^2\,k_{p4}$$
$$-k_{21}^2\,k_{41}^2\,k_{31}\,k_{p4} + k_{21}^2\,k_{41}\,k_{31}^2\,k_{p3} + k_{21}^2\,k_{e2}\,k_{31}^2\,k_{p3}$$
$$-k_{21}^2\,k_{41}^2\,k_{e2}\,k_{p4} + k_{21}^2\,k_{p4}\,k_{41}\,k_{31}^2 + k_{p4}\,k_{41}^3\,k_{e2}^2$$
$$-k_{21}\,k_{31}^2\,k_{41}\,k_{p2}\,k_{e2} - k_{21}\,k_{31}\,k_{41}\,k_{p2}\,k_{e2}^2 + k_{21}\,k_{e2}\,k_{31}^2\,k_{p4}\,k_{41}$$
$$+k_{21}^2\,k_{e2}\,k_{31}\,k_{p4}\,k_{41} + k_{21}\,k_{e2}^2\,k_{p4}\,k_{41}\,k_{31} - k_{21}\,k_{41}\,k_{e2}^2\,k_{p3}\,k_{31}$$
$$-k_{p3}\,k_{21}^2\,k_{31}\,k_{41}\,k_{e2} - 2\,k_{21}\,k_{41}^2\,k_{31}\,k_{p4}\,k_{e2})) - x_4(t) = 0$$

$$C_{3,t} = (2\,k_{21}\,k_{41}^2\,k_{e1}\,(\frac{d}{dt}\,x_1(t))\,k_{p4}\,k_{e2} + k_{41}^2\,k_{e2}^2\,k_{e1}\,(\frac{d}{dt}\,x_1(t))\,k_{p4} \tag{3}$$
$$+k_{41}\,k_{p4}\,k_{e2}^3\,k_{e1}\,(\frac{d}{dt}\,x_1(t)) + k_{21}^2\,k_{p4}\,k_{41}^2\,k_{e1}\,(\frac{d}{dt}\,x_1(t))$$
$$+k_{21}^2\,k_{p4}\,k_{41}\,k_{e2}\,k_{e1}\,(\frac{d}{dt}\,x_1(t)) + k_{21}^2\,k_{p2}\,k_{e2}^2\,k_{e1}\,(\frac{d}{dt}\,x_1(t))$$
$$+k_{21}^2\,k_{p3}\,k_{31}^2\,k_{e1}\,(\frac{d}{dt}\,x_1(t)) + k_{21}^2\,k_{p3}\,k_{31}\,k_{e2}\,k_{e1}\,(\frac{d}{dt}\,x_1(t))$$
$$+k_{31}\,k_{p3}\,k_{e2}^3\,k_{e1}\,(\frac{d}{dt}\,x_1(t)) - k_{31}^2\,k_{e2}^2\,k_{e1}\,(\frac{d}{dt}\,x_1(t))\,k_{p3}$$
$$+k_{21}\,k_{p2}\,k_{e2}^3\,k_{e1}\,(\frac{d}{dt}\,x_1(t)) + k_{41}^2\,k_{e2}^2\,(\frac{d^2}{dt^2}\,x_1(t))\,k_{p4}$$

$$+k_{41}\,k_{p4}\,k_{e2}^3\,(\frac{d^2}{dt^2}\,x_1(t)) + k_{41}\,(\frac{d^4}{dt^4}\,x_1(t))\,k_{p4}\,k_{e2}$$
$$+k_{21}^2\,k_{p2}\,k_{e2}\,(\frac{d^3}{dt^3}\,x_1(t)) + k_{21}^2\,k_{p2}\,k_{e2}^2\,(\frac{d^2}{dt^2}\,x_1(t))$$
$$+k_{21}\,k_{p2}\,(\frac{d^4}{dt^4}\,x_1(t))\,k_{e2} + k_{21}\,k_{p2}\,k_{e2}^3\,(\frac{d^2}{dt^2}\,x_1(t))$$
$$+k_{31}\,k_{p3}\,k_{e2}^3\,(\frac{d^2}{dt^2}\,x_1(t)) + k_{31}\,(\frac{d^4}{dt^4}\,x_1(t))\,k_{p3}\,k_{e2}$$
$$-2\,k_{31}^2\,(\frac{d^3}{dt^3}\,x_1(t))\,k_{p3}\,k_{e2} - k_{31}^2\,k_{e2}^2\,(\frac{d^2}{dt^2}\,x_1(t))\,k_{p3}$$
$$+k_{21}^2\,k_{p4}\,k_{41}^2\,(\frac{d^2}{dt^2}\,x_1(t)) + k_{21}^2\,k_{p4}\,k_{41}\,(\frac{d^3}{dt^3}\,x_1(t))$$
$$+k_{21}^2\,k_{p3}\,k_{31}\,(\frac{d^3}{dt^3}\,x_1(t)) + k_{21}^2\,k_{p3}\,k_{31}^2\,(\frac{d^2}{dt^2}\,x_1(t))$$
$$+k_{21}\,k_{41}\,k_{p4}\,(\frac{d^4}{dt^4}\,x_1(t)) + k_{21}\,k_{p3}\,k_{31}\,(\frac{d^4}{dt^4}\,x_1(t))$$
$$+k_{41}^2\,(\frac{d^3}{dt^3}\,x_1(t))\,k_{p4}\,k_{e2} + 2\,k_{41}\,k_{p4}\,k_{e2}^2\,(\frac{d^3}{dt^3}\,x_1(t))$$
$$+2\,k_{21}\,k_{p2}\,k_{e2}^2\,(\frac{d^3}{dt^3}\,x_1(t)) + 2\,k_{31}\,k_{p3}\,k_{e2}^2\,(\frac{d^3}{dt^3}\,x_1(t))$$
$$-k_{e2}^2\,k_{p3}\,k_{31}^3\,(\frac{d}{dt}\,x_1(t)) + 2\,k_{21}\,k_{e2}^2\,k_{e1}\,(\frac{d}{dt}\,x_1(t))\,k_{p4}\,k_{41}$$
$$+2\,k_{21}\,k_{e2}^2\,k_{e1}\,(\frac{d}{dt}\,x_1(t))\,k_{p3}\,k_{31} + k_{21}\,k_{e1}\,(\frac{d}{dt}\,x_1(t))\,k_{31}^2\,k_{p3}\,k_{e2}$$
$$+k_{21}^2\,k_{p4}\,k_{41}\,k_{e1}\,(\frac{d^2}{dt^2}\,x_1(t)) + k_{21}^2\,k_{p4}\,k_{41}\,k_{e2}\,(\frac{d^2}{dt^2}\,x_1(t))$$
$$+k_{21}^2\,k_{p2}\,k_{e2}\,k_{e1}\,(\frac{d^2}{dt^2}\,x_1(t)) + k_{21}\,k_{p2}\,k_{e1}\,(\frac{d^3}{dt^3}\,x_1(t))\,k_{e2}$$
$$+k_{21}^2\,k_{p3}\,k_{31}\,k_{e2}\,(\frac{d^2}{dt^2}\,x_1(t)) + k_{21}^2\,k_{p3}\,k_{31}\,k_{e1}\,(\frac{d^2}{dt^2}\,x_1(t))$$
$$+k_{21}\,k_{41}\,k_{p4}\,k_{e1}\,(\frac{d^3}{dt^3}\,x_1(t)) + 2\,k_{21}\,k_{41}^2\,(\frac{d^2}{dt^2}\,x_1(t))\,k_{p4}\,k_{e2}$$
$$+k_{31}\,k_{e1}\,(\frac{d^3}{dt^3}\,x_1(t))\,k_{p3}\,k_{e2} - 2\,k_{31}^2\,k_{e1}\,(\frac{d^2}{dt^2}\,x_1(t))\,k_{p3}\,k_{e2}$$
$$+k_{41}\,k_{e1}\,(\frac{d^3}{dt^3}\,x_1(t))\,k_{p4}\,k_{e2} + k_{21}\,k_{p3}\,k_{31}\,k_{e1}\,(\frac{d^3}{dt^3}\,x_1(t))$$
$$+3\,k_{21}\,(\frac{d^3}{dt^3}\,x_1(t))\,k_{p4}\,k_{e2}\,k_{41} + 3\,k_{21}\,(\frac{d^3}{dt^3}\,x_1(t))\,k_{p3}\,k_{e2}\,k_{31}$$
$$+2\,k_{21}\,k_{e2}^2\,(\frac{d^2}{dt^2}\,x_1(t))\,k_{p3}\,k_{31} + 2\,k_{21}\,k_{e2}^2\,(\frac{d^2}{dt^2}\,x_1(t))\,k_{p4}\,k_{41}$$
$$+3\,k_{21}\,k_{e1}\,(\frac{d^2}{dt^2}\,x_1(t))\,k_{p4}\,k_{e2}\,k_{41} + 3\,k_{21}\,k_{e1}\,(\frac{d^2}{dt^2}\,x_1(t))\,k_{p3}\,k_{e2}\,k_{31}$$
$$+2\,k_{21}\,k_{p2}\,k_{e2}^2\,k_{e1}\,(\frac{d^2}{dt^2}\,x_1(t)) + 2\,k_{31}\,k_{p3}\,k_{e2}^2\,k_{e1}\,(\frac{d^2}{dt^2}\,x_1(t))$$
$$+k_{21}\,(\frac{d^2}{dt^2}\,x_1(t))\,k_{31}^2\,k_{p3}\,k_{e2} + k_{41}^2\,k_{e1}\,(\frac{d^2}{dt^2}\,x_1(t))\,k_{p4}\,k_{e2}$$
$$+2\,k_{41}\,k_{p4}\,k_{e2}^2\,k_{e1}\,(\frac{d^2}{dt^2}\,x_1(t)) + k_{41}^2\,k_{e2}\,k_{e1}\,x_1(t)\,k_{31}^2\,k_{p3}$$
$$-k_{21}\,k_{e2}^2\,(\frac{d}{dt}\,x_1(t))\,k_{41}\,k_{p3}\,k_{31} + k_{21}\,k_{e2}^2\,(\frac{d}{dt}\,x_1(t))\,k_{31}^2\,k_{p3}$$

$$+k_{41}\,k_{e1}\,x_1(t)\,k_{31}^2\,k_{e2}^2\,k_{p3}+k_{21}^2\,k_{41}^2\,k_{p2}\,k_{e1}\,(\frac{d}{dt}\,x_1(t))$$
$$-k_{21}\,k_{p3}\,k_{31}^3\,k_{e2}\,k_{e1}\,x_1(t)-k_{21}\,k_{p3}\,k_{31}^3\,k_{41}\,k_{e1}\,x_1(t)$$
$$+k_{21}^2\,k_{p3}\,k_{31}^2\,k_{e2}\,(\frac{d}{dt}\,x_1(t))+k_{21}^2\,k_{p3}\,k_{31}^2\,k_{41}\,(\frac{d}{dt}\,x_1(t))$$
$$+k_{21}^2\,k_{p3}\,k_{31}^2\,k_{41}\,k_{e1}\,x_1(t)-k_{21}^2\,k_{p3}\,k_{31}\,k_{e2}\,(\frac{d}{dt}\,x_1(t))\,k_{41}$$
$$-k_{21}^2\,k_{41}^2\,k_{e1}\,x_1(t)\,k_{p3}\,k_{31}-k_{21}^2\,k_{41}^2\,(\frac{d}{dt}\,x_1(t))\,k_{p3}\,k_{31}$$
$$+k_{31}\,k_{41}^2\,k_{e1}\,(\frac{d}{dt}\,x_1(t))\,k_{p3}\,k_{e2}-k_{21}\,k_{e2}\,(\frac{d}{dt}\,x_1(t))\,k_{41}^2\,k_{p3}\,k_{31}$$
$$+k_{21}\,k_{41}^2\,k_{e1}\,x_1(t)\,k_{31}^2\,k_{p3}+k_{21}\,k_{41}\,k_{e1}\,(\frac{d}{dt}\,x_1(t))\,k_{p3}\,k_{e2}\,k_{31}$$
$$+k_{21}^2\,k_{p3}\,k_{31}^2\,k_{e2}\,k_{e1}\,x_1(t)-k_{21}^2\,k_{p3}\,k_{31}\,k_{41}\,k_{e2}\,k_{e1}\,x_1(t)$$
$$-k_{21}\,k_{p3}\,k_{31}^3\,k_{41}\,(\frac{d}{dt}\,x_1(t))-k_{21}\,k_{p3}\,k_{31}^3\,k_{e1}\,(\frac{d}{dt}\,x_1(t))$$
$$+k_{21}\,k_{e1}\,(\frac{d}{dt}\,x_1(t))\,k_{31}^2\,k_{41}\,k_{p3}+k_{31}^2\,k_{41}\,k_{e1}\,(\frac{d}{dt}\,x_1(t))\,k_{p3}\,k_{e2}$$
$$+k_{31}^2\,k_{41}^2\,k_{p3}\,k_{e1}\,(\frac{d}{dt}\,x_1(t))+k_{21}\,k_{41}^2\,(\frac{d}{dt}\,x_1(t))\,k_{31}^2\,k_{p3}$$
$$-k_{21}\,k_{e2}^2\,k_{e1}\,x_1(t)\,k_{41}\,k_{p3}\,k_{31}+k_{21}\,k_{41}^2\,k_{e2}\,k_{p2}\,k_{e1}\,(\frac{d}{dt}\,x_1(t))$$
$$-k_{21}\,k_{p3}\,k_{31}^3\,k_{e2}\,(\frac{d}{dt}\,x_1(t))-k_{p3}\,k_{31}^3\,k_{41}\,k_{e2}\,(\frac{d}{dt}\,x_1(t))$$
$$+k_{21}\,k_{e2}^2\,k_{e1}\,x_1(t)\,k_{31}^2\,k_{p3}-k_{21}\,k_{41}^2\,k_{e2}\,k_{e1}\,x_1(t)\,k_{p3}\,k_{31}$$
$$+k_{41}\,(\frac{d}{dt}\,x_1(t))\,k_{31}^2\,k_{e2}^2\,k_{p3}+k_{e2}\,(\frac{d}{dt}\,x_1(t))\,k_{41}^2\,k_{31}^2\,k_{p3}$$
$$-k_{e2}^2\,k_{p3}\,k_{31}^3\,k_{e1}\,x_1(t)-k_{p3}\,k_{31}^3\,k_{41}\,k_{e2}\,k_{e1}\,x_1(t)$$
$$-k_{p3}\,k_{31}^3\,k_{41}\,k_{e1}\,(\frac{d}{dt}\,x_1(t))-k_{p3}\,k_{31}^2\,k_{e1}\,(\frac{d^3}{dt^3}\,x_1(t))$$
$$-k_{p3}\,k_{31}^3\,k_{e1}\,(\frac{d^2}{dt^2}\,x_1(t))+k_{21}^2\,k_{41}^2\,k_{p2}\,(\frac{d^2}{dt^2}\,x_1(t))$$
$$+k_{21}\,k_{41}^2\,k_{p2}\,(\frac{d^3}{dt^3}\,x_1(t))+k_{21}\,k_{41}\,k_{p2}\,(\frac{d^4}{dt^4}\,x_1(t))$$
$$+k_{31}\,k_{41}^2\,k_{p3}\,(\frac{d^3}{dt^3}\,x_1(t))+k_{31}\,k_{41}\,k_{p3}\,(\frac{d^4}{dt^4}\,x_1(t))$$
$$+k_{31}^2\,k_{41}^2\,k_{p3}\,(\frac{d^2}{dt^2}\,x_1(t))+k_{21}^2\,k_{p2}\,(\frac{d^3}{dt^3}\,x_1(t))\,k_{41}$$
$$+k_{21}\,k_{41}^2\,k_{p4}\,(\frac{d^3}{dt^3}\,x_1(t))-k_{21}\,k_{p3}\,k_{31}^3\,(\frac{d^2}{dt^2}\,x_1(t))$$
$$-k_{p3}\,k_{31}^3\,k_{41}\,(\frac{d^2}{dt^2}\,x_1(t))-2\,(\frac{d^2}{dt^2}\,x_1(t))\,k_{31}^3\,k_{p3}\,k_{e2}$$
$$+2\,k_{21}\,k_{41}\,k_{e1}\,x_1(t)\,k_{31}^2\,k_{p3}\,k_{e2}+2\,k_{21}^2\,k_{41}\,k_{e2}\,k_{p2}\,k_{e1}\,(\frac{d}{dt}\,x_1(t))$$
$$+2\,k_{31}\,k_{41}\,k_{e2}^2\,k_{e1}\,(\frac{d}{dt}\,x_1(t))\,k_{p3}+2\,k_{21}\,k_{41}\,(\frac{d}{dt}\,x_1(t))\,k_{31}^2\,k_{p3}\,k_{e2}$$
$$+2\,k_{21}\,k_{41}\,k_{p2}\,k_{e2}^2\,k_{e1}\,(\frac{d}{dt}\,x_1(t))-2\,k_{e1}\,(\frac{d}{dt}\,x_1(t))\,k_{31}^3\,k_{p3}\,k_{e2}$$
$$-k_{p3}\,k_{31}^2\,(\frac{d^4}{dt^4}\,x_1(t))-k_{p3}\,k_{31}^3\,(\frac{d^3}{dt^3}\,x_1(t))+k_{21}\,k_{41}^2\,k_{p2}\,k_{e1}\,(\frac{d^2}{dt^2}\,x_1(t))$$

$$
\begin{aligned}
&+k_{21}\,(\frac{d^3}{dt^3}\,x_1(t))\,k_{41}\,k_{p3}\,k_{31}+k_{21}\,k_{41}^2\,k_{p4}\,k_{e1}\,(\frac{d^2}{dt^2}\,x_1(t))\\
&+k_{31}\,k_{41}^2\,(\frac{d^2}{dt^2}\,x_1(t))\,k_{p3}\,k_{e2}+k_{31}\,k_{41}^2\,k_{p3}\,k_{e1}\,(\frac{d^2}{dt^2}\,x_1(t))\\
&+k_{31}\,k_{41}\,k_{p3}\,k_{e1}\,(\frac{d^3}{dt^3}\,x_1(t))+k_{21}\,k_{e2}\,(\frac{d^2}{dt^2}\,x_1(t))\,k_{41}\,k_{p3}\,k_{31}\\
&+k_{21}\,k_{e1}\,(\frac{d^2}{dt^2}\,x_1(t))\,k_{41}\,k_{p3}\,k_{31}+k_{21}^2\,k_{p2}\,k_{e1}\,(\frac{d^2}{dt^2}\,x_1(t))\,k_{41}\\
&+k_{31}^2\,k_{41}\,(\frac{d^2}{dt^2}\,x_1(t))\,k_{p3}\,k_{e2}+k_{21}\,(\frac{d^2}{dt^2}\,x_1(t))\,k_{31}^2\,k_{41}\,k_{p3}\\
&+k_{21}\,k_{41}^2\,k_{e2}\,k_{p2}\,(\frac{d^2}{dt^2}\,x_1(t))+k_{21}\,k_{41}\,k_{p2}\,k_{e1}\,(\frac{d^3}{dt^3}\,x_1(t))\\
&+2\,k_{21}^2\,k_{41}\,k_{e2}\,k_{p2}\,(\frac{d^2}{dt^2}\,x_1(t))+2\,k_{31}\,k_{41}\,k_{e2}^2\,(\frac{d^2}{dt^2}\,x_1(t))\,k_{p3}\\
&+3\,k_{31}\,k_{41}\,k_{e1}\,(\frac{d^2}{dt^2}\,x_1(t))\,k_{p3}\,k_{e2}+3\,k_{31}\,k_{41}\,(\frac{d^3}{dt^3}\,x_1(t))\,k_{p3}\,k_{e2}\\
&+3\,k_{21}\,k_{41}\,k_{p2}\,k_{e2}\,(\frac{d^3}{dt^3}\,x_1(t))+3\,k_{21}\,k_{41}\,k_{p2}\,k_{e2}\,k_{e1}\,(\frac{d^2}{dt^2}\,x_1(t))\\
&+2\,k_{21}\,k_{41}\,k_{p2}\,k_{e2}^2\,(\frac{d^2}{dt^2}\,x_1(t)))/(k_{31}(k_{31}^2\,k_{41}^2\,k_{p4}\,k_{e2}\\
&-k_{21}^2\,k_{41}\,k_{p2}\,k_{e2}^2+k_{21}^2\,k_{31}\,k_{p2}\,k_{e2}^2-k_{21}^2\,k_{41}^2\,k_{p2}\,k_{e2}\\
&+k_{21}^2\,k_{31}^2\,k_{41}\,k_{p2}-k_{21}^2\,k_{31}\,k_{41}^2\,k_{p2}+k_{21}^2\,k_{31}^2\,k_{p2}\,k_{e2}\\
&+k_{21}\,k_{31}^2\,k_{41}^2\,k_{p2}+k_{31}^2\,k_{41}\,k_{e2}^2\,k_{p3}+k_{31}^2\,k_{41}^2\,k_{e2}\,k_{p3}\\
&+k_{21}\,k_{41}^2\,k_{31}^2\,k_{p4}+k_{21}\,k_{e2}^2\,k_{p3}\,k_{31}^2+k_{31}\,k_{41}^2\,k_{e2}^2\,k_{p4}\\
&+k_{21}\,k_{41}^2\,k_{31}^2\,k_{p3}-k_{21}^2\,k_{41}^2\,k_{p3}\,k_{31}-k_{21}\,k_{41}^2\,k_{e2}^2\,k_{p4}\\
&-k_{21}^2\,k_{41}^2\,k_{31}\,k_{p4}+k_{21}^2\,k_{41}\,k_{31}^2\,k_{p3}+k_{21}^2\,k_{e2}\,k_{31}^2\,k_{p3}\\
&-k_{21}^2\,k_{41}^2\,k_{e2}\,k_{p4}+k_{21}^2\,k_{p4}\,k_{41}\,k_{31}^2-k_{p3}\,k_{31}^3\,k_{e2}^2\\
&+k_{21}\,k_{31}\,k_{41}\,k_{p2}\,k_{e2}^2+k_{21}^2\,k_{e2}\,k_{31}\,k_{p4}\,k_{41}+k_{21}\,k_{e2}^2\,k_{p4}\,k_{41}\,k_{31}\\
&-k_{21}\,k_{41}\,k_{e2}^2\,k_{p3}\,k_{31}-k_{p3}\,k_{21}^2\,k_{31}\,k_{41}\,k_{e2}+k_{21}\,k_{31}\,k_{41}^2\,k_{p2}\,k_{e2}\\
&-k_{21}\,k_{p2}\,k_{31}^3\,k_{41}-k_{21}\,k_{p2}\,k_{31}^3\,k_{e2}-k_{21}\,k_{p3}\,k_{31}^3\,k_{41}\\
&-k_{21}\,k_{p3}\,k_{31}^3\,k_{e2}-k_{21}\,k_{31}^3\,k_{p4}\,k_{41}-k_{21}\,k_{31}^2\,k_{e2}^2\,k_{p2}\\
&-k_{p3}\,k_{31}^3\,k_{41}\,k_{e2}-k_{31}^3\,k_{p4}\,k_{41}\,k_{e2}-k_{31}^2\,k_{p4}\,k_{e2}^2\,k_{41}\\
&-k_{21}\,k_{41}^2\,k_{e2}\,k_{p3}\,k_{31}+2\,k_{p3}\,k_{21}\,k_{31}^2\,k_{41}\,k_{e2}))-x_3(t)=0
\end{aligned}
$$

$$
\begin{aligned}
C_{4,t}=&(-k_{41}^2\,k_{e2}^2\,k_{e1}\,(\frac{d}{dt}\,x_1(t))\,k_{p4}-k_{41}\,k_{p4}\,k_{e2}^3\,k_{e1}\,(\frac{d}{dt}\,x_1(t))\\
&+k_{21}^2\,k_{p2}\,k_{e2}^2\,k_{e1}\,(\frac{d}{dt}\,x_1(t))-k_{21}^2\,k_{31}^2\,k_{p2}\,k_{e1}\,(\frac{d}{dt}\,x_1(t))\\
&-2\,k_{31}\,k_{41}^2\,k_{e1}\,(\frac{d}{dt}\,x_1(t))\,k_{p4}\,k_{e2}-k_{31}\,k_{p3}\,k_{e2}^3\,k_{e1}\,(\frac{d}{dt}\,x_1(t))\\
&-k_{31}^2\,k_{p4}\,k_{41}^2\,k_{e1}\,(\frac{d}{dt}\,x_1(t))-k_{31}^2\,k_{p4}\,k_{41}\,k_{e2}\,k_{e1}\,(\frac{d}{dt}\,x_1(t))\\
&-k_{31}^2\,k_{e2}^2\,k_{e1}\,(\frac{d}{dt}\,x_1(t))\,k_{p3}-k_{21}\,k_{31}^2\,k_{e2}\,k_{p2}\,k_{e1}\,(\frac{d}{dt}\,x_1(t))\\
&-k_{21}\,k_{p2}\,k_{e2}^3\,k_{e1}\,(\frac{d}{dt}\,x_1(t))-k_{41}^2\,k_{e2}^2\,(\frac{d^2}{dt^2}\,x_1(t))\,k_{p4}
\end{aligned}
\tag{4}
$$

$$-k_{41}\,k_{p4}\,k_{e2}^3\,(\frac{d^2}{dt^2}\,x_1(t)) - k_{41}\,(\frac{d^4}{dt^4}\,x_1(t))\,k_{p4}\,k_{e2}$$
$$-k_{21}^2\,k_{31}^2\,k_{p2}\,(\frac{d^2}{dt^2}\,x_1(t)) + 2\,k_{21}^2\,k_{p2}\,k_{e2}\,(\frac{d^3}{dt^3}\,x_1(t))$$
$$+k_{21}^2\,k_{p2}\,k_{e2}^2\,(\frac{d^2}{dt^2}\,x_1(t)) - k_{21}\,k_{31}\,k_{p2}\,(\frac{d^4}{dt^4}\,x_1(t))$$
$$-k_{21}\,k_{31}^2\,k_{p2}\,(\frac{d^3}{dt^3}\,x_1(t)) - k_{21}\,k_{p2}\,(\frac{d^4}{dt^4}\,x_1(t))\,k_{e2}$$
$$-k_{21}\,k_{p2}\,k_{e2}^3\,(\frac{d^2}{dt^2}\,x_1(t)) - k_{31}\,k_{41}\,k_{p4}\,(\frac{d^4}{dt^4}\,x_1(t))$$
$$-k_{31}\,k_{p3}\,k_{e2}^3\,(\frac{d^2}{dt^2}\,x_1(t)) - k_{31}\,(\frac{d^4}{dt^4}\,x_1(t))\,k_{p3}\,k_{e2}$$
$$-k_{31}^2\,(\frac{d^3}{dt^3}\,x_1(t))\,k_{p3}\,k_{e2} - k_{31}^2\,k_{e2}^2\,(\frac{d^2}{dt^2}\,x_1(t))\,k_{p3}$$
$$-k_{31}^2\,k_{p4}\,k_{41}^2\,(\frac{d^2}{dt^2}\,x_1(t)) - k_{31}^2\,k_{p4}\,k_{41}\,(\frac{d^3}{dt^3}\,x_1(t))$$
$$-k_{41}^2\,(\frac{d^3}{dt^3}\,x_1(t))\,k_{p4}\,k_{e2} - 2\,k_{41}\,k_{p4}\,k_{e2}^2\,(\frac{d^3}{dt^3}\,x_1(t))$$
$$-2\,k_{21}\,k_{p2}\,k_{e2}^2\,(\frac{d^3}{dt^3}\,x_1(t)) - 2\,k_{31}\,k_{p3}\,k_{e2}^2\,(\frac{d^3}{dt^3}\,x_1(t))$$
$$-k_{21}^2\,k_{31}\,k_{e2}\,k_{p2}\,k_{e1}\,(\frac{d}{dt}\,x_1(t)) - 2\,k_{31}\,k_{41}\,k_{e2}^2\,k_{e1}\,(\frac{d}{dt}\,x_1(t))\,k_{p4}$$
$$-2\,k_{21}\,k_{31}\,k_{p2}\,k_{e2}^2\,k_{e1}\,(\frac{d}{dt}\,x_1(t)) + 2\,k_{21}^2\,k_{p2}\,k_{e2}\,k_{e1}\,(\frac{d^2}{dt^2}\,x_1(t))$$
$$-k_{21}\,k_{p2}\,k_{e1}\,(\frac{d^3}{dt^3}\,x_1(t))\,k_{e2} - k_{21}\,k_{31}^2\,k_{p2}\,k_{e1}\,(\frac{d^2}{dt^2}\,x_1(t))$$
$$-k_{21}\,k_{31}^2\,k_{e2}\,k_{p2}\,(\frac{d^2}{dt^2}\,x_1(t)) - 2\,k_{31}\,k_{41}^2\,(\frac{d^2}{dt^2}\,x_1(t))\,k_{p4}\,k_{e2}$$
$$-k_{31}\,k_{41}\,k_{p4}\,k_{e1}\,(\frac{d^3}{dt^3}\,x_1(t)) - k_{31}\,k_{e1}\,(\frac{d^3}{dt^3}\,x_1(t))\,k_{p3}\,k_{e2}$$
$$-k_{31}^2\,k_{p4}\,k_{41}\,k_{e1}\,(\frac{d^2}{dt^2}\,x_1(t)) - k_{31}^2\,k_{p4}\,k_{41}\,k_{e2}\,(\frac{d^2}{dt^2}\,x_1(t))$$
$$-k_{31}^2\,k_{e1}\,(\frac{d^2}{dt^2}\,x_1(t))\,k_{p3}\,k_{e2} - k_{41}\,k_{e1}\,(\frac{d^3}{dt^3}\,x_1(t))\,k_{p4}\,k_{e2}$$
$$-k_{21}\,k_{31}\,k_{p2}\,k_{e1}\,(\frac{d^3}{dt^3}\,x_1(t)) - k_{21}^2\,k_{31}\,k_{e2}\,k_{p2}\,(\frac{d^2}{dt^2}\,x_1(t))$$
$$-2\,k_{21}\,k_{p2}\,k_{e2}^2\,k_{e1}\,(\frac{d^2}{dt^2}\,x_1(t)) - 2\,k_{21}\,k_{31}\,k_{p2}\,k_{e2}^2\,(\frac{d^2}{dt^2}\,x_1(t))$$
$$-3\,k_{21}\,k_{31}\,k_{p2}\,k_{e2}\,(\frac{d^3}{dt^3}\,x_1(t)) - 2\,k_{31}\,k_{41}\,k_{e2}^2\,(\frac{d^2}{dt^2}\,x_1(t))\,k_{p4}$$
$$-3\,k_{31}\,k_{41}\,(\frac{d^3}{dt^3}\,x_1(t))\,k_{p4}\,k_{e2} - 3\,k_{31}\,k_{41}\,k_{e1}\,(\frac{d^2}{dt^2}\,x_1(t))\,k_{p4}\,k_{e2}$$
$$-2\,k_{31}\,k_{p3}\,k_{e2}^2\,k_{e1}\,(\frac{d^2}{dt^2}\,x_1(t)) - k_{41}^2\,k_{e1}\,(\frac{d^2}{dt^2}\,x_1(t))\,k_{p4}\,k_{e2}$$
$$-2\,k_{41}\,k_{p4}\,k_{e2}^2\,k_{e1}\,(\frac{d^2}{dt^2}\,x_1(t)) - 3\,k_{21}\,k_{31}\,k_{p2}\,k_{e2}\,k_{e1}\,(\frac{d^2}{dt^2}\,x_1(t))$$
$$-k_{21}^2\,k_{41}^2\,k_{p2}\,k_{e1}\,(\frac{d}{dt}\,x_1(t)) - k_{31}\,k_{41}^2\,k_{e1}\,(\frac{d}{dt}\,x_1(t))\,k_{p3}\,k_{e2}$$
$$-2\,k_{31}^2\,k_{41}\,k_{e1}\,(\frac{d}{dt}\,x_1(t))\,k_{p3}\,k_{e2} - k_{31}^2\,k_{41}^2\,k_{p3}\,k_{e1}\,(\frac{d}{dt}\,x_1(t))$$

$$-k_{21}\,k_{41}^2\,k_{e2}\,k_{p2}\,k_{e1}\,(\frac{d}{dt}\,x_1(t)) - k_{21}^2\,k_{41}^2\,k_{p2}\,(\frac{d^2}{dt^2}\,x_1(t))$$
$$-k_{21}\,k_{41}^2\,k_{p2}\,(\frac{d^3}{dt^3}\,x_1(t)) - k_{21}\,k_{41}\,k_{p2}\,(\frac{d^4}{dt^4}\,x_1(t))$$
$$-k_{31}\,k_{41}^2\,k_{p3}\,(\frac{d^3}{dt^3}\,x_1(t)) - k_{31}\,k_{41}\,k_{p3}\,(\frac{d^4}{dt^4}\,x_1(t))$$
$$-k_{31}^2\,k_{41}^2\,k_{p3}\,(\frac{d^2}{dt^2}\,x_1(t)) + k_{21}^3\,k_{e2}^2\,k_{p2}\,(\frac{d}{dt}\,x_1(t))$$
$$-k_{21}^2\,k_{41}\,k_{e2}\,k_{p2}\,k_{e1}\,(\frac{d}{dt}\,x_1(t)) - 2\,k_{31}\,k_{41}\,k_{e2}^2\,k_{e1}\,(\frac{d}{dt}\,x_1(t))\,k_{p3}$$
$$-2\,k_{21}\,k_{41}\,k_{p2}\,k_{e2}^2\,k_{e1}\,(\frac{d}{dt}\,x_1(t)) - k_{21}\,k_{41}^2\,k_{p2}\,k_{e1}\,(\frac{d^2}{dt^2}\,x_1(t))$$
$$-k_{31}\,k_{41}^2\,(\frac{d^2}{dt^2}\,x_1(t))\,k_{p3}\,k_{e2} - k_{31}\,k_{41}^2\,k_{p3}\,k_{e1}\,(\frac{d^2}{dt^2}\,x_1(t))$$
$$-k_{31}\,k_{41}\,k_{p3}\,k_{e1}\,(\frac{d^3}{dt^3}\,x_1(t)) - 2\,k_{31}^2\,k_{41}\,(\frac{d^2}{dt^2}\,x_1(t))\,k_{p3}\,k_{e2}$$
$$-k_{21}\,k_{41}^2\,k_{e2}\,k_{p2}\,(\frac{d^2}{dt^2}\,x_1(t)) - k_{21}\,k_{41}\,k_{p2}\,k_{e1}\,(\frac{d^3}{dt^3}\,x_1(t))$$
$$-k_{21}^2\,k_{41}\,k_{e2}\,k_{p2}\,(\frac{d^2}{dt^2}\,x_1(t)) - 2\,k_{31}\,k_{41}\,k_{e2}^2\,(\frac{d^2}{dt^2}\,x_1(t))\,k_{p3}$$
$$-3\,k_{31}\,k_{41}\,k_{e1}\,(\frac{d^2}{dt^2}\,x_1(t))\,k_{p3}\,k_{e2} - 3\,k_{31}\,k_{41}\,(\frac{d^3}{dt^3}\,x_1(t))\,k_{p3}\,k_{e2}$$
$$-3\,k_{21}\,k_{41}\,k_{p2}\,k_{e2}\,(\frac{d^3}{dt^3}\,x_1(t)) - 3\,k_{21}\,k_{41}\,k_{p2}\,k_{e2}\,k_{e1}\,(\frac{d^2}{dt^2}\,x_1(t))$$
$$-2\,k_{21}\,k_{41}\,k_{p2}\,k_{e2}^2\,(\frac{d^2}{dt^2}\,x_1(t)) + k_{21}^3\,k_{p2}\,k_{e1}\,(\frac{d}{dt}\,x_1(t))\,k_{31}$$
$$+k_{21}^3\,k_{p2}\,k_{41}\,(\frac{d}{dt}\,x_1(t))\,k_{31} + k_{21}^3\,k_{e2}\,k_{p2}\,(\frac{d}{dt}\,x_1(t))\,k_{41}$$
$$+k_{21}^3\,k_{p2}\,k_{41}\,k_{e1}\,(\frac{d}{dt}\,x_1(t)) + k_{21}^3\,k_{41}\,k_{e2}\,k_{p2}\,k_{e1}\,x_1(t)$$
$$+k_{21}^3\,k_{p2}\,k_{41}\,k_{e1}\,x_1(t)\,k_{31} + k_{21}^3\,k_{e2}^2\,k_{p2}\,k_{e1}\,x_1(t)$$
$$+k_{21}^3\,k_{e2}\,k_{p2}\,(\frac{d}{dt}\,x_1(t))\,k_{31} + k_{21}^3\,k_{e2}\,k_{p2}\,k_{e1}\,x_1(t)\,k_{31}$$
$$-k_{21}^2\,k_{41}\,k_{e2}^2\,k_{p2}\,k_{e1}\,x_1(t) - k_{21}^2\,k_{41}^2\,k_{p2}\,k_{e2}\,k_{e1}\,x_1(t)$$
$$+k_{21}^3\,k_{p2}\,k_{e1}\,(\frac{d^2}{dt^2}\,x_1(t)) + k_{21}^3\,k_{p2}\,k_{41}\,(\frac{d^2}{dt^2}\,x_1(t))$$
$$+k_{21}^3\,k_{p2}\,(\frac{d^2}{dt^2}\,x_1(t))\,k_{31} - k_{21}^2\,k_{31}\,k_{p2}\,k_{41}\,k_{e1}\,(\frac{d}{dt}\,x_1(t))$$
$$-k_{21}^2\,k_{41}^2\,k_{p2}\,k_{e2}\,(\frac{d}{dt}\,x_1(t)) - k_{21}^2\,k_{41}\,k_{e2}^2\,k_{p2}\,(\frac{d}{dt}\,x_1(t))$$
$$-k_{21}^2\,k_{31}\,k_{e2}^2\,k_{p2}\,k_{e1}\,x_1(t) - k_{21}^2\,k_{31}\,k_{41}^2\,k_{p2}\,k_{e1}\,x_1(t)$$
$$-k_{21}^2\,k_{31}^2\,k_{41}\,k_{p2}\,k_{e1}\,x_1(t) - k_{21}^2\,k_{31}^2\,k_{p2}\,k_{e2}\,k_{e1}\,x_1(t)$$
$$-k_{21}^2\,k_{31}\,k_{41}^2\,k_{p2}\,(\frac{d}{dt}\,x_1(t)) - k_{21}^2\,k_{31}\,k_{e2}^2\,k_{p2}\,(\frac{d}{dt}\,x_1(t))$$
$$-k_{21}^2\,k_{31}^2\,k_{41}\,k_{p2}\,(\frac{d}{dt}\,x_1(t)) - k_{21}^2\,k_{31}^2\,k_{p2}\,k_{e2}\,(\frac{d}{dt}\,x_1(t))$$
$$-k_{21}\,k_{31}\,k_{41}\,k_{e2}\,k_{p2}\,k_{e1}\,(\frac{d}{dt}\,x_1(t)) + k_{21}\,k_{31}\,k_{41}\,k_{e2}^2\,k_{p2}\,k_{e1}\,x_1(t)$$

$$+k_{21}\,k_{31}\,k_{41}^2\,k_{p2}\,k_{e2}\,(\frac{d}{dt}\,x_1(t))+k_{21}\,k_{31}\,k_{41}\,k_{e2}^2\,k_{p2}\,(\frac{d}{dt}\,x_1(t))$$
$$+k_{21}\,k_{31}\,k_{41}^2\,k_{p2}\,k_{e2}\,k_{e1}\,x_1(t)+k_{21}\,k_{31}^2\,k_{e2}\,k_{p2}\,(\frac{d}{dt}\,x_1(t))\,k_{41}$$
$$+k_{21}\,k_{31}^2\,k_{41}\,k_{e2}\,k_{p2}\,k_{e1}\,x_1(t)+k_{21}\,k_{31}^2\,k_{41}^2\,k_{p2}\,k_{e1}\,x_1(t)$$
$$+k_{21}\,k_{31}^2\,k_{41}^2\,k_{p2}\,(\frac{d}{dt}\,x_1(t))+2\,k_{21}^3\,k_{e2}\,k_{p2}\,k_{e1}\,(\frac{d}{dt}\,x_1(t))$$
$$-2\,k_{21}^2\,k_{31}\,k_{e2}\,k_{p2}\,(\frac{d}{dt}\,x_1(t))\,k_{41}-2\,k_{21}^2\,k_{31}\,k_{41}\,k_{e2}\,k_{p2}\,k_{e1}\,x_1(t)$$
$$+k_{21}^2\,k_{p2}\,(\frac{d^4}{dt^4}\,x_1(t))+k_{21}^3\,k_{p2}\,(\frac{d^3}{dt^3}\,x_1(t))+k_{21}^2\,k_{p2}\,k_{e1}\,(\frac{d^3}{dt^3}\,x_1(t))$$
$$-k_{31}\,k_{41}^2\,k_{p4}\,(\frac{d^3}{dt^3}\,x_1(t))-k_{31}^2\,k_{41}\,k_{p3}\,(\frac{d^3}{dt^3}\,x_1(t))$$
$$+2\,k_{21}^3\,k_{e2}\,k_{p2}\,(\frac{d^2}{dt^2}\,x_1(t))-k_{21}^2\,k_{31}\,k_{p2}\,k_{41}\,(\frac{d^2}{dt^2}\,x_1(t))$$
$$-k_{31}\,k_{41}^2\,k_{p4}\,k_{e1}\,(\frac{d^2}{dt^2}\,x_1(t))-k_{31}^2\,k_{41}\,k_{p3}\,k_{e1}\,(\frac{d^2}{dt^2}\,x_1(t))$$
$$-k_{21}\,k_{31}\,k_{41}\,k_{p2}\,k_{e1}\,(\frac{d^2}{dt^2}\,x_1(t))-k_{21}\,k_{31}\,k_{41}\,k_{e2}\,k_{p2}\,(\frac{d^2}{dt^2}\,x_1(t))$$
$$-k_{21}\,k_{31}\,k_{41}\,k_{p2}\,(\frac{d^3}{dt^3}\,x_1(t)))/(k_{21}(k_{31}^2\,k_{41}^2\,k_{p4}\,k_{e2}$$
$$-k_{21}^2\,k_{41}\,k_{p2}\,k_{e2}^2-k_{21}^2\,k_{31}\,k_{p2}\,k_{e2}^2-k_{21}^2\,k_{41}^2\,k_{p2}\,k_{e2}$$
$$-k_{21}^2\,k_{31}^2\,k_{41}\,k_{p2}-k_{21}^2\,k_{31}\,k_{41}^2\,k_{p2}-k_{21}^2\,k_{31}^2\,k_{p2}\,k_{e2}$$
$$+k_{21}\,k_{31}^2\,k_{41}^2\,k_{p2}+k_{31}^2\,k_{41}\,k_{e2}^2\,k_{p3}+k_{31}^2\,k_{41}^2\,k_{e2}\,k_{p3}$$
$$+k_{21}\,k_{41}^2\,k_{31}^2\,k_{p4}-k_{21}\,k_{e2}^2\,k_{p3}\,k_{31}^2+k_{31}\,k_{41}^2\,k_{e2}^2\,k_{p4}$$
$$+k_{21}\,k_{41}^2\,k_{31}^2\,k_{p3}-k_{21}^2\,k_{41}^2\,k_{p3}\,k_{31}-k_{21}\,k_{41}^2\,k_{e2}^2\,k_{p4}$$
$$-k_{21}^2\,k_{41}^2\,k_{31}\,k_{p4}-k_{21}^2\,k_{41}\,k_{31}^2\,k_{p3}-k_{21}^2\,k_{e2}\,k_{31}^2\,k_{p3}$$
$$-k_{21}^2\,k_{41}^2\,k_{e2}\,k_{p4}-k_{21}^2\,k_{p4}\,k_{41}\,k_{31}^2+k_{21}\,k_{31}^2\,k_{41}\,k_{p2}\,k_{e2}$$
$$+k_{21}\,k_{31}\,k_{41}\,k_{p2}\,k_{e2}^2-k_{21}\,k_{e2}\,k_{31}^2\,k_{p4}\,k_{41}-k_{21}\,k_{e2}^2\,k_{p4}\,k_{41}\,k_{31}$$
$$-k_{21}\,k_{41}\,k_{e2}^2\,k_{p3}\,k_{31}+k_{21}^3\,k_{p2}\,k_{e2}^2+k_{21}\,k_{31}\,k_{41}^2\,k_{p2}\,k_{e2}$$
$$-k_{21}\,k_{41}^2\,k_{e2}\,k_{p3}\,k_{31}+k_{21}^3\,k_{31}\,k_{p2}\,k_{e2}+k_{21}^3\,k_{41}\,k_{p2}\,k_{e2}$$
$$+k_{21}^3\,k_{31}\,k_{41}\,k_{p2}+k_{21}^2\,k_{p4}\,k_{e2}^2\,k_{41}+k_{21}^2\,k_{p3}\,k_{e2}^2\,k_{31}$$
$$+k_{21}^3\,k_{e2}\,k_{p4}\,k_{41}+k_{21}^3\,k_{e2}\,k_{p3}\,k_{31}+k_{21}^3\,k_{41}\,k_{p3}\,k_{31}$$
$$+k_{21}^3\,k_{p4}\,k_{41}\,k_{31}-2\,k_{21}^2\,k_{31}\,k_{41}\,k_{p2}\,k_{e2}))-x_2(t)=0$$

$$C_{5,t}=-(\frac{d^3}{dt^3}\,x_1(t))\,k_{21}\,k_{41}-(\frac{d^4}{dt^4}\,x_1(t))\,k_{e1}-(\frac{d^4}{dt^4}\,x_1(t))\,k_{21} \quad (5)$$
$$-(\frac{d^4}{dt^4}\,x_1(t))\,k_{31}-(\frac{d^4}{dt^4}\,x_1(t))\,k_{41}-(\frac{d^2}{dt^2}\,x_1(t))\,k_{e2}^3-3\,(\frac{d^3}{dt^3}\,x_1(t))\,k_{e2}^2$$
$$-3\,(\frac{d^4}{dt^4}\,x_1(t))\,k_{e2}-k_{e1}\,(\frac{d}{dt}\,x_1(t))\,k_{e2}^3-(\frac{d^2}{dt^2}\,x_1(t))\,k_{41}\,k_{e2}^2$$
$$-k_{e1}\,(\frac{d}{dt}\,x_1(t))\,k_{21}\,k_{e2}^2-k_{e1}\,(\frac{d}{dt}\,x_1(t))\,k_{41}\,k_{e2}^2$$
$$-k_{e1}\,(\frac{d}{dt}\,x_1(t))\,k_{31}\,k_{e2}^2-k_{e1}\,(\frac{d^2}{dt^2}\,x_1(t))\,k_{21}\,k_{41}$$

$$
\begin{aligned}
&-k_{e1}\,(\frac{d^2}{dt^2}\,x_1(t))\,k_{21}\,k_{31}-k_{e1}\,(\frac{d^2}{dt^2}\,x_1(t))\,k_{41}\,k_{31}\\
&-(\frac{d^2}{dt^2}\,x_1(t))\,k_{41}\,k_{31}\,k_{21}-(\frac{d^2}{dt^2}\,x_1(t))\,k_{41}\,k_{e2}\,k_{21}\\
&-(\frac{d^2}{dt^2}\,x_1(t))\,k_{31}\,k_{e2}\,k_{21}-(\frac{d^2}{dt^2}\,x_1(t))\,k_{41}\,k_{e2}\,k_{31}\\
&-2\,k_{e1}\,(\frac{d^2}{dt^2}\,x_1(t))\,k_{21}\,k_{e2}-2\,k_{e1}\,(\frac{d^2}{dt^2}\,x_1(t))\,k_{41}\,k_{e2}\\
&-2\,k_{e1}\,(\frac{d^2}{dt^2}\,x_1(t))\,k_{31}\,k_{e2}-k_{e1}\,(\frac{d}{dt}\,x_1(t))\,k_{31}\,k_{e2}\,k_{21}\\
&-k_{e1}\,(\frac{d}{dt}\,x_1(t))\,k_{41}\,k_{e2}\,k_{31}-k_{e1}\,(\frac{d}{dt}\,x_1(t))\,k_{41}\,k_{31}\,k_{21}\\
&-k_{e1}\,(\frac{d}{dt}\,x_1(t))\,k_{41}\,k_{e2}\,k_{21}-k_{e1}\,(\frac{d^3}{dt^3}\,x_1(t))\,k_{31}-k_{e1}\,(\frac{d^3}{dt^3}\,x_1(t))\,k_{21}\\
&-k_{e1}\,(\frac{d^3}{dt^3}\,x_1(t))\,k_{41}-(\frac{d^2}{dt^2}\,x_1(t))\,k_{21}\,k_{e2}^2-(\frac{d^2}{dt^2}\,x_1(t))\,k_{31}\,k_{e2}^2\\
&-(\frac{d^3}{dt^3}\,x_1(t))\,k_{21}\,k_{31}-(\frac{d^3}{dt^3}\,x_1(t))\,k_{41}\,k_{31}-3\,k_{e1}\,(\frac{d^2}{dt^2}\,x_1(t))\,k_{e2}^2\\
&-3\,k_{e1}\,(\frac{d^3}{dt^3}\,x_1(t))\,k_{e2}-2\,(\frac{d^3}{dt^3}\,x_1(t))\,k_{21}\,k_{e2}-2\,(\frac{d^3}{dt^3}\,x_1(t))\,k_{41}\,k_{e2}\\
&-2\,(\frac{d^3}{dt^3}\,x_1(t))\,k_{31}\,k_{e2}-\frac{d^5}{dt^5}\,x_1(t)=0
\end{aligned}
$$
